# Factors Associated with Childhood Stunting in Four North African Countries: Evidence from Multiple Indicator Cluster Surveys, 2014–2019

**DOI:** 10.3390/nu16040473

**Published:** 2024-02-06

**Authors:** Nagwa Farag Elmighrabi, Catharine A. K. Fleming, Kingsley E. Agho

**Affiliations:** 1 School of Health Science, Western Sydney University, Campbelltown, NSW 2560, Australiak.agho@westernsydney.edu.au (K.E.A.); 2 Department of People Determination and Sustainable Development, Benghazi 18251, Libya; 3 Department of Nutrition, Faculty of Public Health, University of Benghazi, Benghazi 18251, Libya; 4 Translational Health Research Institute (THRI), School of Medicine, Western Sydney University, Penrith, NSW 2750, Australia; 5 Faculty of Health Sciences, University of Johannesburg, Johannesburg 2094, South Africa

**Keywords:** undernutrition, factors, Algeria, Egypt, Sudan, Tunisia

## Abstract

Stunting remains a significant public health issue among North African children, even though significant progress has been made in reducing hunger and poverty. This study aimed to identify factors associated with stunting among children in four North African countries (Algeria, Egypt, Sudan, and Tunisia). A logistic regression model adjusted for clustering and sampling weights was used to identify factors associated with childhood stunting. It was found that the prevalence of stunting in Algeria, Egypt, Sudan, and Tunisia was 9.7%(95% CI: 9.1, 10.3), 21.1% (95% CI: 19.8, 22.5), 33.8% (95% CI: 32.7, 34.9), and 8.2% (95% CI: 7.3, 9.2), respectively. Stunting was more common among children from Sudan and Egypt. Our analysis showed that a low wealth index, being a boy, low BMI, dietary diversity <5 foods, and low birth weight were associated with stunting from 0 to 23 months; however, rural residency, a low-educated mother, low BMI, family size, and diarrhea were associated with stunting from 24 to 59 months. A collaborative approach that prioritizes maternal health and nutrition, invests in struggling families, and customizes interventions to meet the specific needs of each North African country is essential for eradicating undernutrition by 2030.

## 1. Introduction

Stunting describes the hindered growth and development observed in children due to factors such as insufficient nutrition, recurring infections, and inadequate psychosocial stimulation [1]. The World Health Organization defines stunting as children whose height is two standard deviations below the Child Growth Standard Median for their age group [1]. Stunting has the potential to impair cognitive and school performance, reduce adult earnings and the capacity for production, and may lead to chronic diseases later in life, especially if it is accompanied by excess weight in childhood [2,3]. Despite progress in some African regions, stunting remains prevalent, with 90% of stunted children in the developing world residing in Africa and Asia and 30.7% living in Africa, which is much higher than the global average of 22.0% [4]. The northern and central parts of Africa have had the greatest drop in stunting [4]. However, North African nations continue to contribute to the high frequency of stunting in Africa [5]. According to recent data from systemic and meta-analysis research, the total pooled prevalence of stunting among children under five in North Africa is around 23.5%, which is greater than the global average of one in five [6]. Even though this region achieved a significant reduction in poverty and hunger during the millennium development era in 2015, there has been only slight progress toward reaching global nutrition targets [4,7].

As a result of child stunting, low- and middle-income countries lose billions of dollars in future revenue, primarily because of lower wages, physical disabilities, and illness-related absences from work [6]. Among Egyptian and Sudanese children, nutritional deficiency costs approximately 1.1 billion Egyptian Pounds (EGP) and 11.66 billion Sudanese Pounds (SDG), respectively. In addition, it is responsible for 11% of deaths among Egyptian children, and expenditures are projected to rise by 32% by 2025, reaching 26.8 billion EGP [8,9]. 

Children in low- and middle-income countries are at greater risk of undernutrition due to a variety of interrelated factors, including their environment, socioeconomic circumstances, lack of access to food, economic difficulties, civil wars, climate change, inequality, poverty, and communicable diseases. Children in North Africa are similarly affected by these determinants, with exacerbating factors such as maternal health, dietary habits, and susceptibility to diseases also playing a part [8,9,10,11,12,13,14,15,16]. In Libya, for instance, children and families are suffering rapid declines in essential public services, particularly education and health services, as a result of conflicts. In addition, food accessibility is reduced because of food shortages, rising food and fuel costs, displacement from homes and means of sustenance, and loss of jobs and incomes [13]. Similarly, the current crisis in Sudan has also been made worse by deteriorating economic conditions, ongoing unrest in various areas, a subpar harvest, and spikes in grain and other food commodity prices around the country [8]. It is therefore important to focus attention on these countries, especially after the WHO 2021 report demonstrated increasing rather than decreasing undernutrition issues in this region [5].

In order to combat stunting in the North African region, several vital efforts, policies, strategies, programs, and interventions are being undertaken at the national and international levels, such as [17] disease prevention and control, growth monitoring and promotion, vitamin A supplementation, water, sanitation and hygiene (WASH) with disease prevention and management, UNICEF efforts with the Libyan government, and advocacy by UN agencies and civil society for child health and protection in Libya during the recent civil conflicts [18]. 

However, there needs to be more up-to-date monitoring and assessment programs to address this problem in almost the entire North African region. There is an agreement by all introduced national or international strategies and policies to invest in data collection, monitoring, and evaluation of nutrition, to generate timely and suitable-quality nutrition information, and to monitor resources and results in order for advocacy to guide program actions, allocate resources, and promote accountability [17,19]. 

Therefore, this research aimed to examine and analyze the most up-to-date information concerning the factors contributing to stunting in children under the age of five in the North African region. To achieve this, this study combined the data from the most recent nationally representative surveys conducted in Algeria, Egypt, Sudan, and Tunisia, four nations in North Africa. The outcomes of this study will provide public health researchers and policymakers in North African areas with critical information that they can use to repurpose nutrition-related resources and programs to better aid families from low socioeconomic backgrounds.

## 2. Materials and Methods

### 2.1. Data Sources

This analysis was based on data collected from the Multiple Indicator Cluster Surveys (MICSs) conducted in four North African countries, which were publicly available. The MICS system, implemented by UNICEF, is an international, cross-sectional data collection system designed to provide internationally comparable information on key health indicators, particularly related to maternal and child health [20]. The survey typically covers the entire country but can also focus on specific regions. To ensure comparability, the MICS surveys employ a standardized complex sampling design, utilizing multistage stratified cluster sampling with defined selection probabilities for each primary unit.

The data used in this study were obtained from the MICS surveys conducted in Algeria (2019), Egypt (2014), Tunisia (2018), and Sudan (2014), respectively. These surveys are accessible on the website: http://mics.unicef.org/ accessed on 6 January 2024. The MICS surveys serve as nationally representative surveys, gathering data on various aspects of health status, including reproductive health, maternal and child health, mortality, nutrition, and self-reported health behavior among adults [21]. Eligible women for data collection were defined as all women aged 15–48 years who were either permanent residents or visitors present in the households on the night before the survey. Information on child health was collected from the mothers, specifically regarding the youngest child aged under five years. Detailed information regarding the sampling design and questionnaire can be found in the country-specific MICS reports [22].

For this study, the analysis encompassed a total of 39,983 children aged 0–59 months across the four North African countries.

### 2.2. Outcome Variable

The outcome variable for this study was stunting, which was determined by the height-for-age Z-score (HAZ). Stunting is an indicator of linear growth retardation and reflects cumulative growth deficits in a child. The HAZ is calculated based on the 2006 WHO growth reference, which compares a child’s height to the median height of a healthy child in the same age group or reference population. It is expressed in terms of the number of standard deviations (SDs) above or below the median height [1].

### 2.3. Potential Covariates

The covariate variables were selected based on the UNICEF conceptual framework of the determinants of nutritional status for maternal and child health [20], which was modified according to a previous study conducted in 35 low–middle-income countries [23]. These covariates were categorized into three main factors:

#### Enabling Factors: Governance, Resources, and Norms (Country, Place of Residence, Urban/Rural)

Underlying factors: socioeconomic factors (child’s age, child’s sex, household wealth quantile, maternal age, paternal age, maternal age at marriage, maternal level of education, maternal marital status, maternal body mass index (BMI), family size, number of children <5, and child’s birth order), healthcare services factors (place of childbirth, type of delivery, delivery assistants, and number of antenatal visits), and household environment factors (sources of drinking water and toilet facilities, type of cooking fuels, and access to media). In the context of household expenses and earnings, the “Household Wealth Quantile” provides a quantitative indicator of a household’s financial condition. Through the Principal Components Analysis (PCA) approach, it has been established as a quantitative representation of household assets. In order to determine a household member’s ranking in the population, a score was assigned to each lawful household member after this measure was computed. There were five wealth categories in this study: poorest, poorer, middle, fourth, and richest at the national level. Poorest and poorer households made up the lowest 40% of households, middle-class households made up the next 20%, and the fourth and richest households made up the top 40% [23].

Immediate factors: dietary intake factors (early initiation of breastfeeding, ever being breastfed, duration of breastfeeding, and dietary diversity) and child health factors (perceived baby size, diarrhea, and cough in the previous two weeks). According to the guidelines for evaluating feeding practices in infants and young children published in 2021, dietary diversity was determined by adding up the variety of foods within the eight food groups that were offered within the past 24 h [24]. These food categories include breast milk, grains (including roots and tubers), legumes and nuts, dairy products such as milk, yogurt, and cheese, animal-based foods such as meat, fish, poultry, and organ meats, eggs, fruits, and vegetables rich in vitamin A, as well as other varieties of fruits and vegetables [24]. A child’s dietary diversity variable was categorized into two categories: those who consumed five or more food groups and those who consumed less than five food groups.

### 2.4. Statistical Analysis

Furthermore, a weighted approach was used to balance the influence of nations with large populations, such as Egypt in 2021, with over 109 million residents, against those with lower populations, such as Tunisia in 2021, with 12.3 million residents [25]. Clustering and stratification techniques based on certain nations were used to achieve this. For tabulating the survey (SVY) results, population-level weights were applied and modified for distinctive, national-specific strata, while clustering was utilized to ascertain proportions and frequency and to determine the rates of stunted children in each nation, along with 95 percent confidence intervals.

To evaluate the degree of stunting, the dependent variable was divided into two categories: category 0 for those who were not stunted (−2SD) and category 1 for those who were stunted (−2SD). Bivariable analyses were employed to explore the association between potential covariates and the study variables. In the pooled dataset analysis, three models were applied: Model 1 incorporated only the enabling factors; Model 2 represented the second stage of analysis and incorporated underlying factors alongside the enabling factors; and in Model 3, the same process was followed in the third stage, where enabling, underlying, and immediate factors were included. This hierarchical multiple regression analysis sought to compare the correlations between several groupings of characteristics while evaluating their associations with stunting in children under the age of five across the three targeted age groups. Furthermore, we conducted a country-specific analysis as reported in Tables S1–S5.

All statistical computations were conducted utilizing STATA/MP Version 17.0 (Stata Corp, College Station, TX, USA). Adjusted odds ratios (AORs) and their corresponding 95% confidence intervals (CIs) were obtained from the adjusted hierarchical multiple logistic regression models to quantify the factors associated with child stunting. The prevalence of stunting was calculated for each country, and an overall prevalence estimate (not weighted by country size) was also calculated for each country.

In our study, we focused on the presence of stunting and factors associated with it in children below age five, specifically focusing on three distinct age categories: 0–23 months, 24–59 months, and 0–59 months. This was crucial because efforts to improve childhood nutrition, encompassing practices like Infant and Young Child Feeding (IYCF), including breastfeeding and complementary feeding practices, along with promoting health awareness and psychosocial stimulation, should be directed toward children who experience stunting during both the periods of 0–23 months and 0–59 months [26].

## 3. Results

This current study included a total of 39,983 children. Characteristics of the study’s weighted and unweighted samples are presented in Table 1, indicating that the weighted and unweighted samples were similar in the pooled dataset. Of this total, 52.5% lived in urban areas. More than half of the children (51.1%) were boys. The different wealth quantile categories for the four countries were almost equally represented in the sample (approximately 20% each). The lowest percentage of children was in the 18–23 months age bracket (9.2%), while the highest percentage was in the 36–41 months age bracket (11.4%). The majority of mothers came from the 20–34 years age bracket (64.1%). A large majority of the mothers (90.5%) were married, and more than 8 out of every 10 (84.8%) had a BMI less than or equal to 18.5 kg/m^2^. Most of the households (66.1%) had between 5 and 10 members, and more than half (54.5%) had two or more children under five. The minority of households used unclean cooking fuels (22.4%), unimproved drinking water (24.9%), and unimproved toilet facilities (45.1%). The majority of households (74.2%) watched television, but less than 30% (26.1) listened to the radio. Most of the children were an average size at birth (62%), did not have diarrhea in the last two weeks before the survey (83.7%), were delivered at a health facility (70.2%), their mothers had between four and seven antenatal visits (45.7%), were delivered with the aid of healthcare experts (82.4%), and were delivered without cesarean section (65.9%). The majority of the children were fed with food from less than five different food groups (70.1%), were breastfed within one hour after delivery (52.4%), were ever breastfed (85.1%), and were breastfed for a period longer than 12 months (53.3%). The number and frequency of potential variables in each country are displayed in Table S1.

### 3.1. Prevalence of Stunting

**Figure 1:**
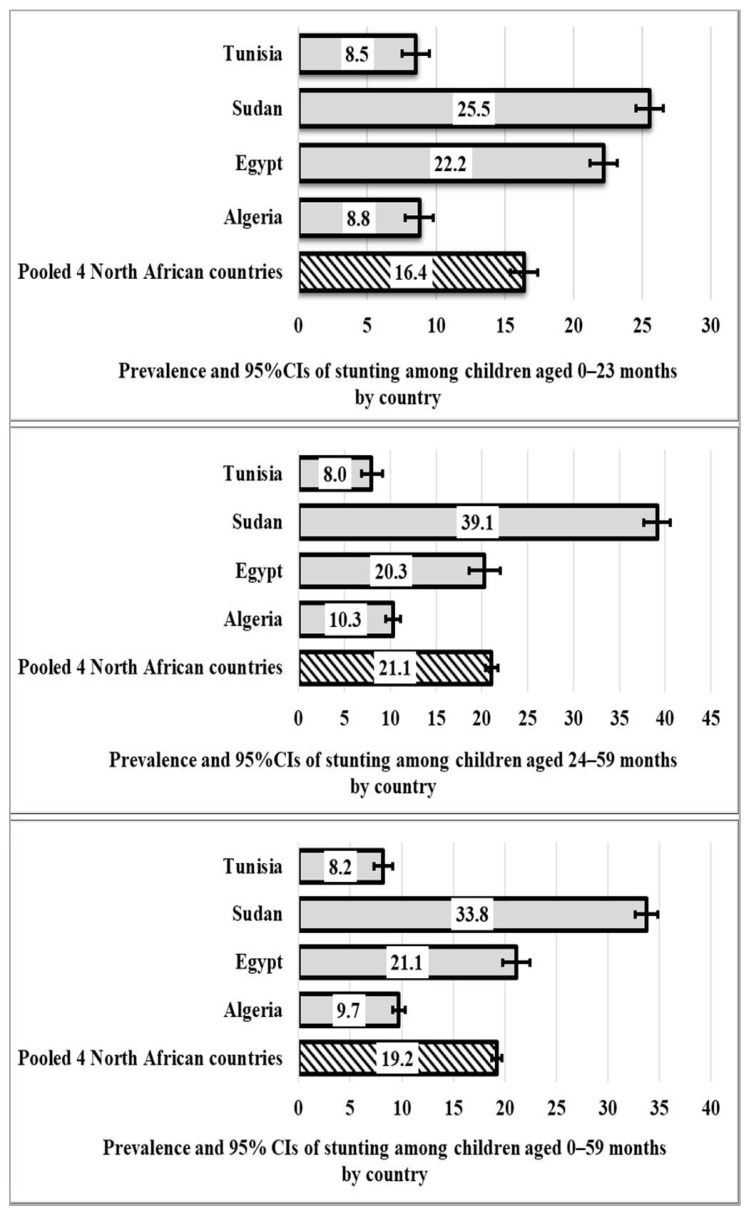
Prevalence and 95% CIs of stunting among children aged (0–23, 24–59, and 0–59 months) in North Africa.

As shown in Figure 1, stunting was most prevalent among children in Sudan (26%) and least prevalent among children in Tunisia (8.5%), with children in Egypt (22%) and Algeria (9%) falling in between. The overall pooled prevalence of stunting among the children aged 0–23 months in the four North African countries was 16.4%.

There was a 39% prevalence of stunting among children aged 24 to 59 months in Sudan. This rate is significantly higher than the combined overall prevalence of 21.1%. The prevalence of stunting was also significant in Egypt (20%), with the lowest rates being in Algeria (10%) and Tunisia (8%), respectively.

In Sudan, children aged 0–59 months exhibited the highest prevalence of stunting at 34%, surpassing the overall pooled prevalence of 19%. Conversely, Tunisia recorded the lowest prevalence of stunting at 8%, while Egypt and Algeria fell in between, with rates of 21% and 10%, respectively.

### 3.2. Multivariate Analysis

Table 2, Table 3 and Table 4 present the pooled prevalence and adjusted odds ratios (aORs) for the association between stunting and potential risk factors among children under five, aged 0–23 months, 24–59 months, and 0–59 months, respectively. Conversely, Table S2 provides a detailed breakdown of the prevalence of this association within each individual country.

#### 3.2.1. Factors Associated with Stunting among Children Aged 0–23 Months

In Table 2, the findings revealed significant associations between stunting among children aged 0–23 months and various factors: children from Egypt or Sudan showed a notable increase in odds of stunting (aOR) and 95% confidence interval (CI): (aOR = 2.88 [95% CI = 1.99, 4.17]) and (aOR = 2.95 [95% CI = 1.90, 4.61]); being from households with a lower wealth index was also significantly associated with stunting (aOR = 1.65 [95% CI = 1.17, 2.34]); children with a low birth weight had a significantly higher likelihood of stunting (aOR = 1.69 [95% CI = 1.42, 2.01]); and a less diverse food intake was found to be significantly associated with stunting (aOR = 1.30 [95% CI = 1.09, 1.55]).

However, decreased odds of stunting among children aged 0–23 months were significantly associated with having a mother whose BMI was less than 18.5 kg/m^2^ (aOR = 0.38 [95% CI = 0.31, 0.47]), older mother (aOR = 0.55 [95% CI = 0.48, 0.91] for 35–49 years), and the child being a girl (aOR = 0.70 [95% CI = 0.60, 0.82]).

In the country-specific analysis (see Table S3), women with a BMI less than 18.5 kg/m^2^ reported lower odds of stunting in Algeria, Egypt, and Tunisia. Children perceived by their mothers to be small reported higher odds of stunting in Sudan, Algeria, Egypt, and Tunisia. Stunting was higher among poorer and poorest households in Sudan, Algeria, Egypt, and Tunisia. Girls reported lower odds of stunting in Sudan, Algeria, and Egypt.

#### 3.2.2. Factors Associated with Stunting among Children Aged 24–59 Months

Table 3 shows that increased odds of stunting among children aged 24–59 months were significantly associated with being from Egypt or Sudan (aOR = 2.64 [95% CI = 1.91, 3.65]) and (aOR = 5.44 [95% CI = 4.13, 7.17]), living in a rural area (aOR = 1.32 [95% CI = 1.13, 1.54]), large family size (aOR = 1.20 [95% CI = 1.04, 1.39]), being ill in the last two weeks (diarrhea) (aOR = 1.28 [95% CI = 1.10, 1.49]), women with a higher BMI and having a mother who had low education (aOR = 1.46 [95% CI = 1.22, 1.75]).

Decreased odds of stunting among children aged 24–59 months were significantly associated with having a mother with a BMI less than 18.5 kg/m^2^ (aOR = 0.24 [95% CI = 0.19, 0.29]), having a father with age 35–44 years (aOR = 0.83 [95% CI = 0.70, 0.98]), being in a household in which the radio was listened to (aOR = 0.82 [95% CI = 0.71, 0.93]), and being in a household in which the television was watched (aOR = 0.69 [95% CI = 0.58, 0.83].

In the country-specific analysis for the 24–59 months age group, women with a BMI less than 18.5 kg/m^2^ reported lower odds of stunting in four North African countries. Compared to the richest, the odds of stunting were higher among poorer and poorest households (see Table S4 for details).

#### 3.2.3. Factors Associated with Stunting among Children Aged 0–59 Months

Increased odds of stunting among children aged 0–59 months were significantly associated with being Sudanese (aOR = 4.20 [3.36, 5.26]) or Egyptian (aOR = 2.80 [95% CI = 2.25, 3.48]), living in a rural area (aOR = 1.25 [95% CI = 1.12, 1.41]), belonging to a poor household (aOR = 1.19 [95% CI = 1.03, 1.37]), being from a large family (aOR = 1.25 [95% CI = 1.01, 1.53]), having a mother with a low education level (aOR = 1.29 [95% CI = 1.11, 1.48]), less diverse food (aOR = 1.21 [95% CI = 1.09, 1.35]), low birthweight (aOR = 1.17 [95% CI = 1.04, 1.32]), and having experienced diarrhea in the last two weeks (aOR = 1.14 [95% CI = 1.03, 1.26]).

Decreased odds of stunting among children aged 0–59 months were significantly associated with being a girl (aOR = 0.83 [95% CI = 0.77, 0.89]), mother with a BMI less than 18.5 kg/m^2^ (aOR = 0.30 [95% CI = 0.26, 0.35]), and belonging to a household with access to the radio (0.84 [076, 0.93]) and/or television (aOR = 0.78 [95% CI = [0.68, 0.90]) (Table 4).

In the country-specific analysis of stunting among children aged 0–59 months, it was found that women with a BMI less than 18.5 kg/m^2^ reported lower odds of stunting in the four countries. Additionally, households with a lower wealth index were associated with stunting in Algeria, Egypt, and Sudan (see Table S5 for details).

**Table 2:** Prevalence and multivariable analysis of stunting among children (0–23 months) in North Africa.

Variables		Model 1		Model 2		Model 3	
	Pr 95%CI	aOR 95%CI	*p* Value	aOR 95%CI	*p* Value	aOR 95%CI	*p* Value
	Enabling factors						
Country							
Algeria		1.00		1.00		1.00	
Egypt		3.20 [2.61, 3.93]	<0.001	3.06 [2.17, 4.32]	<0.001	2.88 [1.99, 4.17]	<0.001
Sudan		3.33 [2.76, 4.02]	<0.001	2.78 [1.90, 4.08]	<0.001	2.95 [1.90, 4.61]	<0.001
Tunisia		0.98 [0.74, 1.30]	0.898	1.22 [0.87, 1.69]	0.245	1.15 [0.82, 1.60]	0.416
Place of residence							
Urban	13.9 [12.8, 15.1]	1.00		1.00		1.00	
Rural	19.1 [17.6, 20.6]	1.26 [1.09, 1.47]	0.002	1.00 [0.81, 1.23]	0.988	0.99 [ 0.80, 1.22]	0.904
	Underlying factors						
	Socioeconomic factors						
Household wealth quantile							
Richest	13 [11.2, 15.0]			1.00		1.00	
Fourth	15.8 [13.9, 17.9]			1.11 [0.87, 1.43]	0.389	1.20 [0.88, 1.44]	0.362
Middle	17 [15.4, 18.9]			1.08 [0.84, 1.40]	0.542	1.10 [0.84, 1.42]	0.488
Poorer	18.2 [16.4, 20.2]			1.14 [0.84, 1.53]	0.405	1.16 [0.87, 1.55]	0.313
Poorest	18.8 [16.6, 21.3]			1.70 [1.20, 2.40]	0.003	1.65 [1.17, 2.34]	0.004
Child’s sex							
Boy	18.8 [17.5, 20.1]			1.00		1.00	
Girl	14 [12.9, 15.1]			0.70 [0.60, 0.82]	<0.001	0.70 [0.60, 0.82]	<0.001
Child’s age							
0–5	11.7 [10.3, 13.3]			1.00		1.00	
6–11	12.4 [11.0, 13.9]			0.73 [0.58, 0.93]	0.012	0.76 [0.59, 0.99]	0.040
12–17	18.5 [16.8, 20.3]			1.32 [1.04, 1.68]	0.024	1.22 [0.79, 1.89]	0.371
18–23	23.1 [21.2, 25.2]			1.63 [1.30, 2.05]	0.000	1.52 [0.92, 2.51]	0.102
Maternal age (years)							
15–19 years	20.8 [17.0, 25.3]			1.00		1.00	
20–34 years	16.7 [15.6, 17.8]			0.68 [0.43, 1.11]	0.127	0.63 [0.39, 1.02]	0.061
35–49 years	15.4 [14.0, 16.9]			0.60 [0.37, 0.98]	0.042	0.55 [0.48, 0.91]	0.019
Paternal age (years)							
18–34	22.1 [20.2, 24.2]			1.00		1.00	
35–44	14.4 [13.2, 15.5]			1.06 [0.84, 1.33]	0.620	1.01 [0.79, 1.28]	0.950
45+	18.7 [16.8, 20.8]			1.16 [0.88, 1.55]	0.294	1.13 [0.84, 1.53]	0.421
Maternal age at marriage							
≤18 years	24.6 [22.6, 26.7]			1.00		1.00	
>18 years	14.4 [13.4, 15.5]			1.15 [0.88, 1.51]	0.294	1.17 [0.88, 1.54]	0.277
Maternal marital status							
Married	17 [16.1, 18.0]			1.00		1.00	
Unmarried *	9.8 [7.7, 12.5]			1.16 [0.11, 12.23]	0.901	1.00 [0.08, 12.89]	0.990
Maternal education							
Secondary and higher	13.8 [12.4, 15.4]			1.00		1.00	
Primary	17 [15.5, 18.7]			1.05 [0.82, 1.33]	0.714	1.05 [0.82, 1.33]	0.717
None	19.2 [17.5, 21.0]			0.85 [0.64, 1.13]	0.269	0.86 (0.72, 1.49)	0.299
Maternal body mass index (BMI)							
18.5–24.9	20.5 [18.3, 22.9]			1.00		1.00	
<18.5	15.2 [14.2, 16.2]			0.39 [0.32, 0.47]	<0.001	0.38 [0.31, 0.47]	<0.001
25+	32.3 [23.2, 43.1]			1.77 [0.89, 3.51]	0.105	1.77 [0.90, 3.48]	0.098
Family size							
2–4	15.1 [13.6, 16.8]			1.00		1.00	
5–10	16.8 [15.7, 18.0]			1.05 [0.85, 1.30]	0.645	1.08 [0.87, 1.35]	0.479
>10	18.1 [14.4, 22.6]			1.20 [0.77, 1.86]	0.421	1.24 [0.79, 1.93]	0.350
Number of children <5	14.3 [13.1, 15.6]						
1	18.1 [17.0, 19.3]			1.00		1.00	
2 or more				1.13 [0.95, 1.34]	0.175	1.16 [0.96, 1.40]	0.125
Child’s birth order							
No previous birth	9.4 [8.1, 10.9]			1.00		1.00	
1	16.9 [15.5, 18.5]			0.98 [0.74, 1.30]	0.877	0.91 [0.68, 1.21]	0.516
2–3	18.3 [16.6, 20.1]			1.05 [0.76, 1.47]	0.737	1.00 [0.72, 1.40]	0.997
4+	27.3 [24.0, 30.8]			1.22 [0.80, 1.88]	0.352	1.13 [0.72, 1.77]	0.607
	Healthcare services						
Number of antenatal visits							
8+	17.3 [15.0, 19.7]			1.00		1.00	
4–7	15.1 [13.9, 16.5]			1.00 [0.83, 1.23]	0.929	1.06 [0.87, 1.30]	0.551
1–3	17.6 [16.0, 19.3]			0.95 [0.72, 1.26]	0.716	0.99 [0.75, 1.32]	0.958
None	17.6 [15.6, 19.8]			1.01 [0.68, 1.50]	0.959	1.06 [0.69, 1.64]	0.792
Place of childbirth							
Health facility	13.6 [12.6, 14.7]			1.00		1.00	
Home	26.4 [24.5, 28.4]			0.68 [0.10, 4.45]	0.686	1.00 [0.19, 5.30]	0.997
Delivery assistants							
Health professionals	16.2 [15.2, 17.3]			1.00		1.00	
Non-health professionals	17.3 [15.3, 19.5]			0.35 [0.65, 1.92]	0.228	0.40 [0.77, 2.08]	0.277
Type of delivery							
Non-cesarean	12.9 [11.7, 14.2]			1.00		1.00	
Cesarean	15.1 [13.5, 16.9]			1.07 [0.90, 1.28]	0.426	1.05 [0.87, 1.26]	0.604
	Household environment						
Type of cooking fuels							
Clean	13.3 [12.3, 14.4]			1.00		1.00	
Unclean	28.2 [26.0, 30.5]			1.36 [0.88, 2.10]	0.185	1.28 [0.82, 2.00]	0.285
Sources of drinking water							
Improved	16.8 [15.8, 18.0]			1.00		1.00	
Unimproved	15 [13.4, 16.8]			0.88 [0.63, 1.23]	0.451	0.86 [0.62, 1.19]	0.366
Type of toilet facility							
Improved	15.2 [13.9, 16.5]			1.00		1.00	
Unimproved	18 [16.7, 19.5]			1.12 [0.83, 1.47]	0.504	1.13 [0.84, 1.52]	0.402
Listening to the radio							
No	16.6 [15.5, 17.8]			1.00		1.00	
Yes	16.3 [14.8, 18.0]			0.87 [0.72, 1.04]	0.126	0.88 [0.73, 1.06]	0.174
Watching TV							
Not at all	26.1 [24.1, 28.2]			1.00		1.00	
Yes	13.4 [12.4, 14.5]			0.99 [0.72, 1.34]	0.917	0.96 [0.70, 1.31]	0.789
	Immediate factors						
	Dietary intake						
Dietary diversity							
5+ food groups	14.1 [12.7, 15.7]					1.00	
<5 food groups	17.4 [16.4, 18.5]					1.30 [1.09, 1.55]	0.004
Early initiation of breastfeeding							
After 1 h	13.4 [12.2, 14.6]					1.00	
Within 1 h	19.3 [18.0, 20.5]					1.00 [0.83, 1.20]	0.993
Ever breastfed							
Yes	17.6 [16.6, 18.7]					1.00	
No	9.7 [8.2, 11.4]					0.92 [0.62, 1.36]	0.674
Duration of breastfeeding							
Up to 12 months	12.9 [11.8, 14.1]					1.00	
>12 months	19.4 [18.1, 20.8]					1.15 [0.76, 1.73]	0.515
	Child health						
Perceived baby size							
Average	15.3 [14.2, 16.5]					1.00	
Small	24.7 [22.7, 26.8]					1.69 [1.42, 2.01]	<0.001
Large	12.5 [10.8, 14.5]					0.62 [0.47, 0.83]	0.001
Diarrhea							
No	15.2 [14.3, 16.3]					1.00	
Yes	20.4 [18.4, 22.6]					0.92 [0.75, 1.12]	0.399
Cough							
No	15.8 [14.7, 17.0]					1.00	
Yes	17.9 [16.3, 19.7]					0.86 [0.71, 1.04]	0.131

Model 1 = basic factors; Model 2 = basic and underlying factors; Model 3 = basic underlying and immediate factors; unmarried * refers to individuals who are single, separated, widowed, or divorced.

**Table 3:** Prevalence and multivariable analysis of stunting among children (24–59 months) in North Africa.

		Model 1		Model 2		Model 3	
Variables	Pr 95% CI	OR 95% CI	*p* Value	OR 95% CI	*p* Value	OR 95% CI	*p* Value
Enabling factors							
Country							
Algeria		1.00		1.00		1.00	
Egypt		2.48 [1.92, 3.20]	<0.001	2.59 [1.88, 3.56]	<0.001	2.64 [1.91, 3.65]	<0.001
Sudan		5.06 [4.37, 5.86]	<0.001	6.06 [4.73, 7.77]	<0.001	5.44 [4.13, 7.17]	<0.001
Tunisia		0.77 [0.60, 0.98]	0.036	0.70 [0.53, 0.92]	0.010	0.68 [0.52, 0.91]	0.008
Place of residence							
Urban	15.2 [13.9, 16.7]	1.00		1.00		1.00	
Rural	27.4 [25.6, 29.4]	1.44 [1.26, 1.65]	<0.001	1.33 [1.13, 1.55]	<0.001	1.32 [1.13, 1.54]	<0.001
Underlying factors							
Socioeconomic factors							
Household wealth quantile							
Richest	18.1 [16.1, 20.4]			1.00		1.00	
Fourth	20.3 [18.2, 22.6]			0.88 [0.67, 1.17]	0.793	0.89 [0.67, 1.18]	0.434
Middle	19.5 [17.7, 21.5]			0.96 [0.76, 1.23]	0.761	0.97 [0.76, 1.23]	0.776
Poorer	24.9 [22.8, 27.1]			1.05 [0.86, 1.29]	0.618	1.04 [0.85, 1.28]	0.686
Poorest	23.1 [21.0, 25.4]			1.17 [0.98, 1.41]	0.073	1.17 [0.98, 1.39]	0.091
Child’s sex							
Boy	21.8 [20.6, 23.1]			1.00		1.00	
Girl	20.3 [18.9, 21.8]			0.93 [0.85, 1.02]	0.122	0.93 [0.85, 1.02]	0.132
Child’s age (months)							
24–29	24.8 [22.7, 26.9]			1.00		1.00	
30–35	24.9 [22.8, 27.1]			1.22 [1.05, 1.43]	0.049	1.24 [1.06, 1.46]	0.006
36–41	19 [17.2, 21.0]			0.70 [0.60, 0.82]	<0.001	0.66 [0.55, 0.79]	<0.001
42–47	24.1 [22.1, 26.2]			1.05 [0.90, 1.22]	0.534	1.00 [0.84, 1.19]	0.981
48–53	16.6 [14.9, 18.5]			0.66 [ 0.56, 0.78]	<0.001	0.62 [0.51, 0.76]	<0.001
54–59	17.2 [15.1, 19.5]			0.73 [0.62, 0.87]	<0.001	0.69 [0.57, 0.84]	<0.001
Maternal age (years)							
15–19 years	16.5 [13.7, 19.8]			1.00		1.00	
20–34 years	22.6 [21.3, 24.1]			1.16 [0.76, 1.75]	0.495	1.19 [0.79, 1.78]	0.415
35–49 years	19 [17.4, 20.7]			1.10 [0.69, 1.76]	0.697	1.13 [0.71, 1.79]	0.619
Paternal age (years)							
18–34	31.6 [28.9, 34.5]			1.00		1.00	
35–44	17.8 [16.7, 19.0]			0.82 [0.70, 0.97]	0.021	0.83 [0.70, 0.98]	0.030
45+	24.1 [22.2, 26.0]			0.84 [0.69, 1.00]	0.060	0.85 [0.70, 1.02]	0.083
Maternal education							
Secondary and higher	15.4 [13.9, 17.1]			1.00		1.00	
Primary	21.7 [20.0, 23.5]			1.31 [1.11, 1.53]	0.001	1.29 [1.10, 1.52]	0.002
None	27.4 [25.5, 29.4]			1.47 [1.23, 1.76]	<0.001	1.46 [1.22, 1.75]	<0.001
Maternal age at marriage							
≤18 years	35.3 [33.1, 37.5]			1.00		1.00	
>18 years	17.2 [16.1, 18.4]			0.96 [ 0.86, 1.08]	0.546	0.96 [ 0.85, 1.08]	0.470
Maternal marital status							
Married	21.8 [20.6, 23.1]			1.00		1.00	
Unmarried *	14.1 [12.0, 16.6]			1.22 [1.04, 1.42]	0.012	0.76 [0.57, 1.03]	0.056
Maternal body mass index (BMI)							
18.5–24.9	32.8 [29.6, 36.2]			1.00		1.00	
<18.5	19.6 [18.3, 20.8]			0.24 [0.19, 0.29]	<0.001	0.24 [0.19, 0.29]	<0.001
25+	35 [26.9, 44.0]			0.84 [0.47, 1.49]	0.555	0.81 [0.45, 1.45]	0.472
Family size							
2–4	17.1 [15.8, 18.5]			1.00		1.00	
5–10	22.2 [20.9, 23.6]			1.20 [1.04, 1.38]	0.013	1.20 [1.04, 1.39]	0.012
>10	24.1 [20.5, 28.1]			1.17 [0.91, 1.50]	0.222	1.16 [0.90, 1.48]	0.250
Number of children < 5							
1	18.4 [17.2, 19.7]			1.00		1.00	
2 or more	23.4 [22.0, 24.9]			0.85 [0.75, 0.96]	0.010	0.86 [0.76, 0.97]	0.015
Child’s birth order							
No previous birth	9.9 [8.6, 11.4]			1.00		1.00	
1	19.2 [17.8, 20.6]			0.83 [0.66, 1.05]	0.115	0.83 [0.66, 1.04]	0.117
2–3	24.8 [23.2, 26.5]			0.83 [0.64, 1.07]	0.141	0.83 [0.64, 1.07]	0.144
4+	33.7 [30.5, 36.9]			0.80 [ 0.61, 1.07]	0.137	0.80 [0.61, 1.07]	0.130
Household environment							
Type of cooking fuels							
Clean	15.6 [14.4, 16.9]			1.00		1.00	
Unclean	41.2 [38.6, 43.8]			1.03 [0.81, 1.31]	0.794	1.05 [0.83, 1.33]	0.678
Sources of drinking water							
Improved	21.5 [20.1, 22.9]			1.00		1.00	
Unimproved	19.9 [18.1, 21.9]			0.99 [0.83, 1.17]	0.889	0.98 [0.82, 1.16]	0.789
Type of toilet facility							
Improved	17.5 [16.2, 19.0]			1.00		1.00	
Unimproved	25.4 [23.7, 27.2]			0.99 [0.83, 1.17]	0.871	1.00 [0.84, 1.19]	0.970
Listening to the radio							
No	21.4 [20.2, 22.7]			1.00		1.00	
Yes	20.7 [19.1, 22.4]			0.82 [0.71, 0.93]	0.003	0.82 [0.71, 0.93]	0.003
Watching TV							
No	39.7 [37.2, 42.2]			1.00		1.00	
Yes	15.2 [14.1, 16.3]			0.69 [0.58, 0.82]	<0.001	0.69 [0.58, 0.83]	<0.001
Immediate factors							
Child health							
Diarrhea							
No	19 [17.9, 20.1]					1.00	
Yes	35.3 [32.3, 38.5]					1.28 [1.10, 1.49]	0.001
Cough							
No	20.4 [19.1, 21.7]					1.00	
Yes	22.8 [21.1, 24.5]					0.93 [0.83, 1.05]	0.226

Model 1 = basic factors; Model 2 = basic and underlying factors; Model 3 = basic underlying and immediate factors; unmarried * refers to individuals who are single, separated, widowed, or divorced.

**Table 4:** Prevalence and multivariable analysis of stunting among children (0–59 months) in North Africa.

		Model 1		Model 2		Model 3	
Variables	Pr 95% CI	OR 95% CI	*p* Value	OR 95% CI	*p* Value	OR 95% CI	*p* Value
		Enabling factors					
Country							
Algeria		1.00		1.00		1.00	
Egypt		2.75 [2.35, 3.22]	<0.001	2.99 [2.42, 3.69]	<0.001	2.80 [2.25, 3.48]	<0.001
Sudan		4.34 [3.84, 4.91]	<0.001	5.27 [4.32, 6.43]	<0.001	4.20 [3.36, 5.26]	<0.001
Tunisia		0.84 [0.69, 1.03]	0.089	0.77 [0.62, 0.97]	0.026	0.77 [0.61, 0.96]	0.003
Place of residence							
Urban	14.7 [13.8, 15.7]	1.00		1.00		1.00	
Rural	24.1 [22.7, 25.6]	1.37 [1.23, 1.52]	<0.001	1.25 [1.11, 1.41]	<0.001	1.25 [1.12, 1.41]	<0.001
		Underlying factors					
		Socioeconomic factors					
Household wealth quantile							
Richest	16.1 [14.6, 17.7]			1.00		1.00	
Fourth	18.4 [16.8, 20.2]			1.02 [0.82, 1.28]	0.835	1.02 [0.82, 1.27]	0.861
Middle	18.6 [17.2, 20.0]			1.07 [0.89, 1.28]	0.499	1.06 [0.89, 1.28]	0.506
Poorer	22.2 [20.5, 23.9]			1.14 [0.98, 1.34]	0.099	1.12 [0.96, 1.31]	0.153
Poorest	21.5 [19.7, 23.4]			1.21 [1.05, 1.40]	0.007	1.19 [1.03, 1.37]	0.015
Child’s sex							
Boy	20.6 [19.6, 21.6]			1.00		1.00	
Girl	17.8 [16.8, 18.8]			0.83 [0.77, 0.89]	<0.001	0.83 [0.77, 0.89]	<0.001
Child’s age (months)							
0–5	11.7 [10.3, 13.3]			1.00		1.00	
6–11	12.4 [11.0, 13.9]			1.03 [0.83, 1.26]	0.808	0.98 [0.80, 1.22]	0.884
12–17	18.5 [16.8, 20.3]			1.97 [1.62, 2.39]	<0.001	1.83 [1.48, 2.25]	<0.001
18–23	23.1 [21.2, 25.2]			3.01 [2.47, 3.66]	<0.001	2.81 [2.27, 3.50]	<0.001
24–29	24.8 [22.7, 26.9]			3.08 [2.49, 3.81]	<0.001	2.89 [2.29, 3.65]	<0.001
30–35	24.9 [22.8, 27.1]			3.70 [2.96, 4.62]	<0.001	3.48 [2.73, 4.45]	<0.001
36–41	19 [17.2, 21.0]			2.17 [1.72, 2.71]	<0.001	1.84 [1.44, 2.34]	<0.001
42–47	24.1 [22.1, 26.2]			3.17 [2.56, 3.93]	<0.001	2.69 [2.13, 3.40]	<0.001
48–53	16.6 [14.9, 18.5]			1.99 [1.58, 2.51]	<0.001	1.68 [1.31, 2.15]	<0.001
54–59	17.2 [15.1, 19.5]			2.19 [1.78, 2.71]	<0.001	1.85 [1.47, 2.33]	<0.001
Maternal age (years)							
15–19 years	18.5 [16.1, 21.2]			1.00		1.00	
20–34 years	20.1 [19.1, 21.1]			0.88 [0.68, 1.13]	0.317	0.86 [0.67, 1.11]	0.246
35–49 years	17.8 [16.6, 19.2]			0.83 [0.62, 1.10]	0.190	0.80 [0.60, 1.07]	0.134
Paternal age (years)							
18–34	27 [25.2, 28.8]			1.00		1.00	
35–44	16.4 [15.5, 17.3]			0.89 [0.78, 1.01]	0.065	0.89 [0.78, 1.01]	0.072
45+	22.3 [20.7, 24.0]			0.93 [0.83, 1.07]	0.292	0.93 [0.80, 1.07]	0.319
Maternal age at marriage							
≤18 years	31 [29.2, 32.7]			1.00		1.00	
>18 years	16.1 [15.3, 16.9]			1.00 [0.91, 1.10]	0.968	0.99 [ 0.90, 1.10]	0.914
Maternal marital status							
Married	19.9 [19.0, 20.8]			1.00		1.00	
Unmarried *	12.7 [11.1, 14.6]			0.72 [0.09, 5.95]	0.754	0.86 [0.09, 8.07]	0.897
Maternal education							
Secondary and higher	14.7 [13.6, 16.0]			1.00		1.00	
Primary	19.9 [18.5, 21.2]			1.20 [1.06, 135]	0.004	1.18 [1.04, 1.34]	0.009
None	24.3 [22.8, 25.9]			1.30 [1.13, 1.49]	<0.001	1.29 [1.11, 1.48]	<0.001
Maternal body mass index (BMI)							
19–25	25.8 [23.8, 28.0]			1.00		1.00	
<=18.5	18 [17.0, 18.9]			0.31 [0.27, 0.35]	<0.001	0.30 [0.26, 0.35]	<0.001
25+	33.9 [27.7, 40.6]			1.05 [0.69, 1.59]	0.826	1.03 [0.86, 1.57]	0.878
Family size							
2–4	16.2 [15.2, 17.3]			1.00		1.00	
5–10	20.2 [19.2, 21.3]			1.19 [1.06, 1.34]	0.003	1.20 [1.06, 1.35]	0.002
>10	21.5 [18.9, 24.4]			1.25 [1.02, 1.53]	0.033	1.25 [1.01, 1.53]	0.032
Number of children < 5							
1	16.8 [15.9, 17.8]			1.00		1.00	
2 or more	21.2 [20.2, 22.4]			0.96 [0.87, 1.05]	0.347	0.96 [0.88, 1.05]	0.384
Child’s birth order							
No previous birth	9.6 [8.6, 10.7]			1.00		1.00	
1	18.3 [17.2, 19.5]			0.87 [0.73, 1.03]	0.108	0.87 [0.73, 1.03]	0.107
2–3	22.4 [21.2, 23.8]			0.85 [0.70, 1.03]	0.107	0.85 [0.70, 1.03]	0.096
4+	31.3 [29.1, 33.7]			0.90 [0.72, 1.12]	0.345	0.89 [0.71, 1.12]	0.291
		Household environment					
Type of cooking fuels							
Clean	14.7 [13.8, 15.6]			1.00		1.00	
Unclean	36.1 [34.0, 38.3]			1.08 [0.92, 1.27]	0.341	1.10 [0.94, 1.30]	0.241
Sources of drinking water							
Improved	19.6 [18.6, 20.7]			1.00		1.00	
UnImproved	18.1 [16.6, 19.7]			1.00 [0.87, 1.16]	0.965	1.00 [0.87, 1.15]	0.986
Type of toilet facility							
Improved	16.6 [15.6, 17.6]			1.00		1.00	
Unimproved	22.6 [21.2, 24.0]			1.00 [0.88, 1.15]	0.955	1.01 [0.89, 1.16]	0.844
Listening to the radio							
No	19.5 [18.6, 20.5]			1.00		1.00	
Yes	19 [17.7, 20.2]			0.84 [0.76, 0.93]	0.001	0.84 [0.76, 0.93]	0.001
Watching TV							
No	34.3 [32.3, 36.4]			1.00		1.00	
Yes	14.5 [13.7, 15.3]			0.78 [0.68, 0.90]	0.001	0.78 [0.68, 0.90]	0.001
		Immediate factors					
		Child health					
Diarrhea							
No	17.6 [16.8, 18.5]					1.00	
Yes	27.4 [25.5, 29.3]					1.14 [1.03, 1.26]	0.013
Cough							
No	18.6 [17.6, 19.6]					1.00	
Yes	20.9 [19.7, 22.1]					0.95 [0.87, 1.03]	0.241

Model 1 = basic factors; Model 2 = basic and underlying factors; Model 3 = basic underlying and immediate factors; unmarried * refers to individuals who are single, separated, widowed, or divorced.

## 4. Discussion

This study focused on investigating the factors associated with stunting in various age groups of children under five in the North African region. The prevalence of stunting in the North African region is critically significant, as indicated by the findings of this study, which employed a combination of nationally representative data from Algeria, Egypt, Sudan, and Tunisia. The region has a higher-than-average rate of stunting, with 19.2% of children aged 0 to 59 experiencing stunting. Notably, the highest prevalence was observed among children aged 24–59 months, reaching 21.1%, which was comparable to the global figure of 21.3% [5]. Our study found a glaring disparity in the prevalence of stunting among the four North African nations, which may be related to disparities in child healthcare, as well as social, economic, and cultural difficulties within and between various populations [27].

Our pooled results explored multiple areas based on the UNICEF conceptual framework categorization, which we adapted based on prior research carried out in low–middle-income countries in order to acquire a deeper knowledge of the variables causing undernutrition in the North African region [23]. A number of important factors were shown to be strongly associated with stunting in this current study, including country type, place of residence, being a girl, wealth quantile, maternal level of education, maternal and paternal age, maternal BMI, family size, dietary diversity, having an illness in the last two weeks, and access to media.

Although a higher BMI in mothers was found to be a factor associated with stunting in the whole region, there was significant variation in country-specific analysis. For instance, in Sudan, a higher BMI in mothers was found to be a protective factor among children under five. However, in Algeria, Egypt, and Tunisia, it was linked to stunting among all age groups. Low birth weight, being a girl, household wealth index, children with older ages, and paternal older ages were found to be factors associated with stunting among children in more than one country.

According to the findings of this study, stunting was more prevalent in Sudan and Egypt than in Algeria and Tunisia among children under five years old in the North African area. It is important to note that none of these countries have achieved SDG 2.2, which aims for zero malnutrition. Additionally, it is concerning that the prevalence of undernutrition in this region is increasing instead of decreasing, highlighting the urgent need for focused attention on this issue both nationally and internationally [5]. Our findings aligned with a recent study on stunting among children under the age of five in five South Asian countries, which also observed an increased likelihood of stunting among children in India [28].

The differences in the probability of stunting and associated factors across the four North African nations can be attributed to unfavorable factors affecting governance and available resources. In the region, these factors play a pivotal role in shaping children’s nutritional well-being. Various obstacles confront North Africa countries that cause a multitude of disparities in malnutrition across the country’s various regions. These challenges encompass political instability, conflicts, poverty, inequality, the impact of climate change, issues of food security, and the prevalence of infectious diseases [11,12,13,14,15,16,29]. These factors collectively affect the nutritional status of individuals, especially children, in the region. Conflicts, for example, disrupt healthcare services and displace populations, leading to overcrowded and unsanitary living conditions, with limited access to healthcare, food, and clean water. Additionally, conflicts cause infrastructure destruction and disrupt supply chains, resulting in food shortages and higher prices, as well as damage to water and sanitation infrastructure, contributing to increased malnutrition among children [10,13,14,30]. It is evident that conflicts have a significant impact on child health [13,15,30,31]. Additionally, climate change disrupts agricultural production and reduces crop yields, leading to food insecurity and malnutrition via its impact on food diversity and availability. Children are particularly vulnerable to nutrient deficiencies, which can result in stunted growth, impaired cognitive development, and weakened immune systems [11]. Libya and Sudan, for example, are among the world’s most water-scarce countries as a direct consequence of climate change [32,33].

Additionally, poverty and inequality are additional potentially harmful elements that heighten these issues by reducing access to diverse and healthy foods and to healthcare and education services [30]. Families living in poverty often struggle to afford a diverse and nutritious diet and face barriers in accessing healthcare services, including prenatal care, immunizations, and timely treatment of illnesses. Additionally, they frequently lack access to sanitary facilities, clean water, and good hygiene habits [16,27]. Inequality in access to education, particularly for children from impoverished backgrounds, can have negative impacts on their cognitive development and overall well-being. Algeria, Tunisia, and Libya already have high rates of poverty and unemployment, and their inequalities are unevenly distributed across age groups, genders, and geographical areas [29]. With Africa being the third most unequal region after Latin America and the Middle East [12,34], it is subject to significant obstacles to achieving the global goal of ending poverty by 2030. Our finding indicated that children who lived in rural areas had significantly higher odds of being stunted compared with those from urban areas, in agreement with results from past studies [28,35,36,37]. In this study, these differences could partly be due to socioeconomic differences in rural and urban settings in these countries [36]. Living in a rural environment and having a mother with lower educational attainment can markedly influence the well-being of a child. A lack of access to healthcare services, including maternity and child healthcare services, in rural areas may delay the delivery of necessary medical care, increasing the danger to the mother and her child [37]. Children’s health in rural areas is further compromised by inadequate sanitation, poor access to nutritious foods, limited educational choices, and inadequate environments [35,37].

According to our study, the mother’s education level plays an important role in determining whether older children in North Africa are at risk of growth impairments. This is consistent with many previous studies, which found an important correlation between maternal education and the level of growth impairment among children [23,28,36,37,38,39]. Mothers with better educational backgrounds demonstrate a significant difference from those with limited or no education. Educated mothers and mothers with a better understanding of nutrition often recognize the importance of early feeding routines. In addition to their awareness of dietary habits and suitable feeding practices, they exhibit greater awareness of the importance of a child’s nutritional health [38]. As a result, they are able to make well-informed decisions regarding their child’s nutrition and general health. Furthermore, educated mothers have a better knowledge and understanding of personal care and sanitation, which are vital for disease prevention and health maintenance. Parents’ potential income can be reduced if they do not have enough education to secure stable employment. The consequences of this may include delayed or inadequate healthcare-seeking behaviors as families prioritize other fundamental needs over healthcare expenditure [38,39,40].

According to our pooled and country-specific findings, females in all three age categories were significantly less likely to experience growth impairment than males, especially in Algeria and Sudan. In the study, boys were found to be more vulnerable to growth impairment. Cultural norms have historically favored girls over boys, contributing to gender-related health disparities [41,42]. Moreover, boys are often involved in a range of physical activities, consuming a considerable amount of energy that might otherwise be used for growth, while cultural norms mean girls are more likely to stay indoors, cook, do household chores, and maintain a less active lifestyle in comparison with boys. Therefore, boys are more likely to suffer from increased caloric and nutritional requirements for optimal growth and development [35]. A number of cross-sectional studies have also found similar results, including in Iran [43], Kenya [44], Indonesia [45], Tanzania [46], and Ghana [47].

Children born to mothers with a higher BMI are more likely to experience stunted growth than those born to mothers with a lower BMI. This finding was consistent with a nationwide cross-sectional study conducted in Ethiopia that found a significant number of mothers in Ethiopia between the ages of 29 and 31 who are overweight or obese have stunted children [48]. Overweight and obese mothers are more likely to stop breastfeeding, which can contribute to their child’s stunted growth and lead to a double burden of malnutrition [49]. This trend may be linked to the fact that obese women are approximately three times less likely to initiate breastfeeding compared to non-obese women [50]. Ensuring adequate breastfeeding plays a crucial role in enhancing the child’s overall nutritional status.

The pooled results of the study exhibited consistency with the country-specific data from Algeria, Egypt, and Tunisia. However, in Sudan, overweight was associated with higher odds of stunting and not obesity. This finding could be attributed to the fact that about three-quarters of mothers in Sudan lived in rural areas, and in developing countries, overweight and obesity are more common in urban areas than in rural areas [51,52].

According to our study, mothers aged 35–49 in this region have a protective effect against stunting among young children. Similarly, fathers aged 35–44 have a protective effect against stunting among children aged 24–59 months. These outcomes are consistent with our specific country analysis conducted in Algeria and Sudan. It is important to note that further research is recommended to determine the exact reasons behind these findings, as they could provide valuable insights for addressing the issue of malnutrition in the region. The cultural factors within these societies might play a role in explaining these effects. One possible explanation is that adolescent mothers may face more challenges in providing adequate nutrition and care for their children due to their own nutritional needs, busy lives, social responsibilities, limited resources, and lack of knowledge and experience. On the other hand, advanced maternal age brings certain advantages, such as increased socioeconomic resources, better knowledge of nutrition, and improved access to healthcare [53]. Furthermore, family support within these societies can help alleviate some of the difficulties faced by mothers [54]. As daughters grow older, they often become more responsible and supportive of their mothers and siblings, which can contribute to better childcare practices and improved nutritional outcomes. While maternal age tends to receive more attention in studies on child malnutrition, paternal age can also influence child nutrition outcomes. Older fathers (usually above 35 years) may possess greater socioeconomic resources and stability and may support the feeding of their babies, which can contribute to better child nutrition [55].

Poverty is positively correlated with childhood growth impairment; children from economically disadvantaged households are more likely to suffer from growth impairment than children from affluent households. The association between wealth and stunting can be attributed to the importance of wealth in acquiring nourishing food and essential goods, which promote and protect children’s health. A number of past studies have demonstrated a positive association between low income and stunting in children [23,28,36,56].

Stunting was found to be positively associated with large family size in our study. Children from larger families were more likely to experience stunting. This can be attributed to the challenges of having many household members, such as limited resources for adequate childcare and lower dietary intake [57]. Undernutrition in children under five can be made worse by having a large family and low income in communities, especially in communities without social security or support services for disadvantaged families. These results were consistent with prior research [58,59] that proved an association between stunting and bigger family sizes. In families with more children, the caregiver-to-child ratio may be higher. This can affect the quality of care and attention given to each child, including feeding practices, hygiene, and stimulation, which are crucial for healthy growth and development [60].

Our pooled results strongly support the significant association between stunting among children in North Africa and insufficient intake of diverse foods, which aligned with the findings of A kombi et al. [35] in their study conducted in the sub-Saharan region. Malnutrition can occur when there is inadequate consumption of macronutrients (proteins, carbohydrates, and fats) and essential micronutrients (vitamins and minerals) [61]. The absence of nutrient-rich foods and inadequate dietary diversity not only leads to undernutrition but also compromises immunity and increases reoccurrence of and susceptibility to illnesses. Furthermore, our study revealed a significant association between stunting in children aged 0–59 months who had experienced diarrhea in the two weeks preceding the surveys. This finding was consistent with previous research indicating that both the cumulative incidence and longitudinal prevalence of diarrhea are strongly linked to stunting prevalence [62]. The persistence or recurrence of diarrhea can lead to depletion of nutrients, decreased nutrient absorption, and increased metabolic demands, thereby contributing to wasting and underweight status. Diarrhea is commonly linked to inadequate hygiene practices, polluted water sources, limited access to medical care, and subpar sanitation facilities [63]. It is imperative to observe that these factors frequently function within a vicious cycle. Insufficient dietary consumption has the potential to undermine a child’s immune system, rendering them more susceptible to infections like diarrhea [62,63,64].

Maternal exposure to radio and/or television was associated with a significantly lower probability of stunting among children aged 0–59 months in our pooled results relative to those whose mothers did not utilize these media. This association was particularly significant in Algeria and Sudan, where maternal exposure to television was linked with a reduced incidence of stunting among children.

Electronic or print media exposure still remains a crucial means of disseminating health-related programs, such as the advantages of breastfeeding, immunization, and complementary feeding [65], thereby making it a vital source of health information.

This study had both strengths and limitations. One of the study’s strengths was its exclusive focus on undernutrition within North Africa, which allowed for a comprehensive analysis of the contributing factors. Through a focus on this particular region, we were able to acquire a more profound understanding of the challenges, circumstances, and contextual factors that contribute to undernutrition in this area. We carefully examined the determinants linked to stunted growth in children under the age of five across three distinct age groups, specifically 0–23 months, 24–59 months, and 0–59 months. Nevertheless, the study had certain limitations; in terms of methodology, the current study’s cross-sectional design restricted our capacity to deduce causation. Additionally, certain data relied on self-reported information, such as the reported birth weight of the children. Furthermore, the outcomes of the study might have been affected by confounding variables that were not thoroughly examined in the analysis.

This study highlighted the pressing need for action and underscored the importance of collaboration between national and international governments and policymakers to prioritize nutrition. It advocated for the application of a comprehensive strategy to tackle undernutrition and involved the creation of individualized plans tailored to the specific context while considering the identified factors that contribute to the problem. One of the strategies that may be employed is the implementation of social safety nets to aid in the access to food in rural areas and regions affected by conflicts. Implementation of multi-year transfers consisting of food, money, or a mixture of both would provide assistance to underprivileged regions [17]. Improved water, sanitation, and hygiene (WASH) in community-based interventions could enhance the protection of children against diarrheal infections. It is imperative to recognize the complex contributing factors to stunting in each country in the region and to develop interventions that are customized to meet the specific needs of each region.

Additionally, this study emphasizes the significance of promoting peace, improving resilience to climate change, reducing poverty, and guaranteeing fair availability of nourishing food. The nutritional status and overall health outcomes of vulnerable populations can be improved by investing in healthcare, education, and social safety nets. Another significant implication of these actions is the creation of evaluation and monitoring systems to measure the effectiveness of interventions and follow progress over time. Consistently collecting and analyzing data can provide insights into intervention impacts, allowing policymakers to adjust strategies and make informed decisions [66].

## 5. Conclusions

In conclusion, the high prevalence of undernutrition stunting in North Africa is a major concern that needs to be addressed. The area is currently subject to various difficulties that include political instability, poverty, disparity, climate change, and pandemics, all of which augment malnutrition and cause food insecurity. Addressing child undernutrition in North Africa requires a comprehensive and multi-sectoral approach tailored to the specific needs of each individual country. Addressing undernutrition among disadvantaged families and areas, particularly maternal health and nutrition, should be the focus of interventions, programs, and policies to effectively tackle this issue. Better healthcare access and parental education on family planning and maternal/child health could make this possible. Combating undernutrition also involves empowering women, enhancing community-based programs, and promoting equitable economic opportunities. A holistic approach that covers these essential aspects could significantly reduce undernutrition and improve children’s overall well-being in the area. 

## Figures and Tables

**Table 1:** Number and frequency of potential variables linked to stunting in North African children.

Variables	N *	% *	%
Enabling factors			
Country			
Algeria	17,139	42.9	43.1
Egypt	5090	12.7	12.3
Sudan	14,081	35.2	35.6
Tunisia	3673	9.2	9.0
Place of residence			
Urban	20,576	52.5	51.3
Rural	19,408	48.5	48.7
Underlying factors			
Socioeconomic factors			
Household wealth quantile			
Poorest	7793	19.5	20.0
Poorer	7378	18.5	20.0
Middle	7918	19.8	20.0
Fourth	8243	20.6	20.0
Richest	8652	21.6	20.0
Child’s sex			
Boy	20,439	51.1	51.2
Girl	19,544	48.9	48.8
Child’s age (months)			
0–5	3986	1.0	1.0
6–11	3929	9.8	9.8
12–17	4211	10.5	10.3
18–23	3679	9.2	9.1
24–29	3950	9.9	10
30–35	3815	9.5	9.6
36–41	4538	11.4	11.4
42–47	4113	10.3	10.3
48–53	4035	10.1	10.2
54–59	3728	9.3	9.3
Maternal age (years)			
15–19 years	1714	4.4	5.0
20–34 years	25,083	64.1	63.9
35–49 years	12,326	31.5	31.2
Paternal age (years)			
18–34	6997	19.5	19.5
35–44	20,219	56.3	56.1
45+	8670	24.2	24.4
Maternal age at marriage			
≤18 years	9373	25.4	25.9
>18 years	27,565	74.6	74.1
Maternal marital status			
Married	35,885	91.7	90.5
Unmarried **	3239	8.3	9.5
Maternal education			
Secondary and higher	12,235	32.4	34.3
Primary	12,364	32.8	31.7
None	13,116	34.8	34
Maternal body mass index (BMI)			
18.5–24.9	5366	14.2	13.7
<18.5	32,098	84.8	85.3
25+	371	1.0	1.1
Family size			
2–4	10,791	27.0	26.5
5–10	26,435	66.1	66.3
>10	2758	6.9	7.2
Number of children <5			
1	18,197	45.5	45.7
2 or more	21,786	54.5	54.4
Child’s birth order			
No previous birth	7963	19.9	19.0
1	15,374	38.5	39.2
2–3	12,225	30.6	31.0
4+	4422	11.1	10.8
Healthcare services			
Place of childbirth			
Health facility	10,119	70.2	69.1
Home	4296	29.8	30.9
Number of antenatal visits			
8+	2293	14.5	14.2
4–7	7226	45.7	44.2
1–3	3394	21.5	22.6
None	2893	18.3	19.0
Delivery assistants			
Health professionals	12,998	82.2	81.1
Non-health professionals	2808	17.8	19.0
Type of delivery			
Non-cesarean	6677	65.9	66.9
Cesarean	3459	34.1	33.1
Household environment			
Type of cooking fuels			
Clean	31,042	77.6	73.9
Unclean	8938	22.4	26.1
Sources of drinking water			
Improved	30,009	75.1	74.1
UnImproved	9947	24.9	25.9
Type of toilet facility			
Improved	21,945	54.9	54.3
Unimproved	17,997	45.1	45.7
Listening to the radio			
No	29,196	73.9	73.2
Yes	10,293	26.1	26.8
Watching TV			
No	10,209	25.8	27.8
Yes	29,294	74.2	72.2
Immediate factors			
Dietary intake			
Dietary diversity			
5+ food groups	4723	29.9	29.0
<5 food groups	11,082	70.1	71.0
Early initiation of breastfeeding			
After 1 h	7528	47.6	46.8
Within 1 h	8277	52.4	53.2
Ever breastfed			
Yes	13,448	85.1	85.4
No	2358	14.9	14.6
Duration of breastfeeding			
Up to 12 months	7385	46.7	47.2
>12 months	8420	53.3	52.8
Child health			
Perceived baby size			
Average	8842	62.0	62.8
Small	3427	24.0	23.3
Large	2004	14.0	13.9
Diarrhea			
No	33,331	83.7	84.9
Yes	6477	16.3	15.1
Cough			
No	28,616	71.7	72.2
Yes	11,292	28.3	27.8

* Weighted number and percentage; ** unmarried refers to individuals who are single, separated, widowed, or divorced.

## Data Availability

Data are available online at https://mics.unicef.org/surveys, accessed on 1 July 2023.

## Supplementary Materials

The following supporting information can be downloaded at: https://www.mdpi.com/article/10.3390/nu16040473/s1, Table S1. Number and frequency of potential variables linked to stunting among children under five, specifically in four North African countries, Algeria, Egypt, Sudan, and Tunisia; Table S2. Prevalence and 95% confidence intervals (CIs) of stunting among children (0–23, 24–59, and 0–59 months) in four North African countries, Algeria 2019 (N = 16,419), Egypt 2014 (N = 5053), Sudan 2014 (N = 12,841), and Tunisia 2018 (N = 3615); Tables S3–S5. Multivariable analysis of stunting among children (0–23 months), (24–59 months), and (0–59 months) in four North African countries, Algeria 2019 (N = 16,419), Egypt 2014 (N = 5053), Sudan 2014 (N = 12,841), and Tunisia 2018 (N = 3615).

## References

[1] De Onis M. (2006). WHO Child Growth Standards: Length/Height-for-Age, Weight-for-Age, Weight-for-Length, Weight-for-Height and Body Mass Index-for-Age.

[2] Scrimshaw N.S., SanGiovanni J.P. (1997). Synergism of nutrition, infection, and immunity: An overview. Am. J. Clin. Nutr..

[3] Black R.E., Allen L.H., Bhutta Z.A., Caulfield L.E., De Onis M., Ezzati M., Mathers C., Rivera J. (2008). Maternal and child undernutrition: Global and regional exposures and health consequences. Lancet.

[4] Country Nutrition Profile: Northern Africa. https://globalnutritionreport.org/resources/nutrition-profiles/africa/northern-africa/.

[5] The United Nations Children’s Fund, World Health Organisation, The World Bank Group (2021). Levels and Trends in Child Malnutrition: UNICEF/WHO/The World Bank Group Joint Child Malnutrition Estimates: Key Findings of the 2021 Edition.

[6] Elmighrabi N.F., Fleming C.A., Dhami M.V., Elmabsout A.A., Agho K.E. (2023). A systematic review and meta-analysis of the prevalence of childhood undernutrition in North Africa. PLoS ONE.

[7] The State of Food Security and Nutrition in the World 2020. https://www.google.com.au/books/edition/The_State_of_Food_Security_and_Nutrition/09zyDwAAQBAJ?hl=en&gbpv=0.

[8] Abu-Fatima O., Abbas A.A.G., Racalbuto V., Smith L., Pizzol D. (2021). Child undernutrition in Sudan: The social and economic impact and future perspectives. Am. J. Trop. Med. Hyg..

[9] The World Food Program The Cost of Hunger in Africa: Egypt 2013. https://www.wfp.org/publications/egypt-cost-hunger-implications-child-undernutrition-social-economic-development-june-2013.

[10] International Crisis Group 10 Conflicts to Watch in 2021. https://www.crisisgroup.org/global/10-conflicts-watch-2021.

[11] How Climate Change Impacts the Economy. https://news.climate.columbia.edu/2019/06/20/climate-change-economy-impacts/.

[12] World Inequality Lab Global Inequality Data 2020 Update. https://wid.world/news-article/2020-regional-updates/#:~:text=The%20World%20Inequality%20Lab%20releases.

[13] Food Security and Nutrition Libya. https://reliefweb.int/report/libya/food-security-and-nutrition-libya-april-2021.

[14] Breisinger C., Van Rheenen T., Ringler C., Pratt A.N., Minot N., Aragon C., Yu B., Ecker O., Zhu T. (2010). Food Security and Economic Development in the Middle East and North Africa: Current State and Future Perspective.

[15] Dahab R., Becares L., Brown M. (2020). Armed conflict as a determinant of children malnourishment: A cross-sectional study in The Sudan. BMC Public Health.

[16] Van de Poel E., Hosseinpoor A.R., Speybroeck N., Van Ourti T., Vega J. (2008). Socioeconomic inequality in malnutrition in developing countries. Bull. World Health Organ..

[17] World Health Organisation Policy National Food & Nutrition Policy & Strategy 2007–2017. https://extranet.who.int/nutrition/gina/en/node/17826.

[18] UNICEF for Every Child Middle East and North Africa. https://www.unicef.org/mena/research-and-reports.

[19] World Health Organisation (2020). Global Action Plan on Child Wasting: A Framework for Action to Accelerate Progress in Preventing and Managing Child Wasting and the Achievement of the Sustainable Development Goals.

[20] United Nations Childre’s Fund (2021). UNICEF Conceptual Framework on Maternal and Child Nutrition.

[21] Khan S., Hancioglu A. (2019). Multiple Indicator Cluster Surveys: Delivering robust data on children and women across the globe. Stud. Fam. Plan..

[22] Multiple Indicator Cluster Surveys: 6 Tools. https://mics.unicef.org/tools?round=mics6.

[23] Li Z., Kim R., Vollmer S., Subramanian S.V. (2020). Factors associated with child stunting, wasting, and underweight in 35 low-and middle-income countries. JAMA Netw. Open.

[24] (2021). Indicators for Assessing Infant and Young Child Feeding Practices: Definitions and Measurement Methods.

[25] Population Total Middle East and North Africa. https://data.worldbank.org/indicator/SP.POP.TOTL?end=2018&locations=ZQ&most_recent_year_desc=true&start=2016.

[26] Daniel A.I., Bandsma R.H., Lytvyn L., Voskuijl W.P., Potani I., van den Heuvel M. (2017). Psychosocial stimulation interventions for children with severe acute malnutrition: A systematic review. J. Glob. Health.

[27] Krishna A., Mejía-Guevara I., McGovern M., Aguayo V.M., Subramanian S.V. (2018). Trends in inequalities in child stunting in South Asia. Matern. Child Nutr..

[28] Wali N., Agho K.E., Renzaho A.M. (2020). Factors associated with stunting among children under 5 years in five South Asian countries (2014–2018): Analysis of demographic health surveys. Nutrients.

[29] Poverty has Fallen in the Maghreb, but Inequality Persists. https://www.worldbank.org/en/news/feature/2016/10/17/poverty-has-fallen-in-the-maghreb-but-inequality-persists#:~:text=Poverty%20and%20unemployment%20rates%20in,ending%20global%20poverty%20by%202030.

[30] World Food Program (2020). 2020 Global Report on Food Crisis.

[31] Sulaiman A.A., Bushara S.O., Elmadhoun W.M., Noor S.K., Abdelkarim M., Aldeen I.N., Osman M.M., Almobarak A.O., Awadalla H., Ahmed M.H. (2018). Prevalence and determinants of undernutrition among children under 5-year-old in rural areas: A cross-sectional survey in North Sudan. J. Fam. Med. Prim. Care.

[32] Water Scarcity and Climate Change: An Analysis on WASH Enabling Environment in Libya. https://www.unicef.org/mena/media/19321/file/Libya%20water%20scarcity%20analysis%20and%20recommendations_%20UNICEF%20Sep%202022.pdf.

[33] Water, Sanitation and Hygiene. https://www.unicef.org/sudan/water-sanitation-and-hygiene.

[34] United Nations (2022). The Sustainable Development Goals Report 2022.

[35] Akombi B.J., Agho K.E., Hall J.J., Wali N., Renzaho A.M., Merom D. (2017). Stunting, wasting and underweight in sub-Saharan Africa: A systematic review. Int. J. Environ. Res. Public Health.

[36] Akombi B.J., Agho K.E., Renzaho A.M., Hall J.J., Merom D.R. (2019). Trends in socioeconomic inequalities in child undernutrition: Evidence from Nigeria Demographic and Health Survey (2003–2013). PLoS ONE.

[37] Chopra M. (2003). Risk factors for undernutrition of young children in a rural area of South Africa. Public Health Nutr..

[38] Lassi Z.S., Das J.K., Zahid G., Imdad A., Bhutta Z.A. (2013). Impact of education and provision of complementary feeding on growth and morbidity in children less than 2 years of age in developing countries: A systematic review. BMC Public Health.

[39] Vikram K., Vanneman R. (2020). Maternal education and the multidimensionality of child health outcomes in India. J. Biosoc. Sci..

[40] Improving Maternal Health Through Education: Safe Motherhood Is a Necessity. https://www.un.org/en/chronicle/article/improving-maternal-health-through-education-safe-motherhood-necessity#:~:text=Mothers%20with%20primary%20education%20tend,earning%20capacity%20is%20better%20understood.

[41] Thurstans S.O.C., Seal A., Wells J., Khara T., Dolan C., Briend A., Myatt M., Garenne M., Sear R., Kerac M. (2020). Boys are more likely to be undernourished than girls: A systematic review and meta-analysis of sex differences in undernutrition. BMJ Glob. Health.

[42] Di Renzo G.C., Rosati A., Sarti R.D., Cruciani L., Cutuli A.M. (2007). Does fetal sex affect pregnancy outcome?. Gend. Med..

[43] Kavosi E., Rostami Z.H., Kavosi Z., Nasihatkon A., Moghadami M., Heidari M. (2014). Prevalence and determinants of under-nutrition among children under six: A cross-sectional survey in Fars province, Iran. Int. J. Health Policy Manag..

[44] Masibo P.K., D (2012). Makoka. Trends and determinants of undernutrition among young Kenyan children: Kenya Demographic and Health Survey; 1993, 1998, 2003 and 2008–2009. Public Health Nutr..

[45] Agho K.E., Inder K.J., Bowe S.J., Jacobs J., Dibley M.J. (2009). Prevalence and risk factors for stunting and severe stunting among under-fives in North Maluku province of Indonesia. BMC Pediatr..

[46] Shiratori S. Determinants of child malnutrition in Tanzania: A quantile regression approach. Proceedings of the Agricultural and Applied Economics Association’s 2014 AAEA Annual Meeting.

[47] Darteh E.K.M., Acquah E., Kumi-Kyereme A. (2014). Correlates of stunting among children in Ghana. BMC Public Health.

[48] Eshete T., Kumera G., Bazezew Y., Marie T., Alemu S., Shiferaw K. (2020). The coexistence of maternal overweight or obesity and child stunting in low-income country: Further data analysis of the 2016 Ethiopia demographic health survey (EDHS). Sci. Afr..

[49] (2006). The association of maternal overweight and obesity with breastfeeding duration. J. Pediatr..

[50] Perez M.R., Castro L.S.A.D., Chang Y.S., Sanudo A., Marcacine K.O., Amir L.H., Ross M.G., Coca K.P. (2021). Breastfeeding practices and problems among obese women compared with nonobese women in a Brazilian hospital. Women’s Health Rep..

[51] Jehn M., Brewis A. (2009). Paradoxical malnutrition in mother–child pairs: Untangling the phenomenon of over-and under-nutrition in underdeveloped economies. Econ. Hum. Biol..

[52] Barquera S., Peterson K.E., Must A.A., Rogers B.L., Flores M., Houser R., Monterrubio E., Rivera-Dommarco J.A. (2007). Coexistence of maternal central adiposity and child stunting in Mexico. Int. J. Obes..

[53] Wemakor A., Garti H., Azongo T., Garti H., Atosona A. (2018). Young maternal age is a risk factor for child undernutrition in Tamale Metropolis, Ghana. BMC Res. Notes.

[54] Fakhr El-Islam M. (2008). Arab culture and mental health care. Transcult. Psychiatry.

[55] Inbaraj L.R., Khaja S., George C.E., Norman G. (2020). Paternal involvement in feeding and its association with nutritional status of children in an urban slum in a low-resource setting: A cross-sectional study. Nutrition.

[56] Reyes H., Pérez-Cuevas R., Sandoval A., Castillo R., Santos J.I., Doubova S.V., Gutiérrez G. (2004). The family as a determinant of stunting in children living in conditions of extreme poverty: A case-control study. BMC Public Health.

[57] Owoaje E., Onifade O., Desmennu A. (2014). Family and socioeconomic risk factors for undernutrition among children aged 6 to 23 months in Ibadan, Nigeri. Pan Afr. Med. J..

[58] Ersino G., Zello G.A., Henry C.J., Regassa N. (2018). Gender and household structure factors associated with maternal and child undernutrition in rural communities in Ethiopia. PLoS ONE.

[59] National Institute of Population Research and Training, Mitra and Associates, ICF International (2013). Bangladesh Demographic and Health Survey 2011.

[60] Miller A.L., Miller S.E., Clark K.M. (2018). Child, caregiver, family, and social-contextual factors to consider when implementing parent-focused child feeding interventions. Curr. Nutr. Rep..

[61] Malnutrition. https://my.clevelandclinic.org/health/diseases/22987-malnutrition.

[62] Checkley W., Buckley G., Gilman R.H., Assis A.M., Guerrant R.L., Morris S.S., Mølbak K., Valentiner-Branth P., Lanata C.F., Black R.E. (2008). Multi-country analysis of the effects of diarrhea on childhood stunting. Int. J. Epidemiol..

[63] Diarrhea. https://www.who.int/news-room/fact-sheets/detail/diarrhoeal-disease.

[64] Bourke C.D., Berkley J.A., Prendergast A.J. (2016). Immune Dysfunction as a Cause and Consequence of Malnutrition. Trends Immunol..

[65] Person B.P.H., Cliff O., Owuor M., Ogange L. Education through Listening: A theory-based, behavior change pedagogy for improving community-level health promoters’ interpersonal communication and community engagement skills in Kenya. Proceedings of the National Conference on Health Communication, Marketing, and Media.

[66] United Nations Children’s Fund (UNICEF) (2020). Nutrition, for Every Child: UNICEF Nutrition Strategy 2020–2030.

