# Supplementary material for: Factors Associated with Childhood Stunting in Four North African Countries: Evidence from Multiple Indicator Cluster Surveys, 2014–2019
Source: Nutrients. 2024 Feb 6;16(4):473. doi: 10.3390/nu16040473 (PMC10892369; doi:10.3390/nu16040473)
Supplement: Supplementary file 1 [file nutrients-16-00473-s001.zip › nutrients-2800450-supplementary.pdf]

Table S1. Number and frequency of potential variables linked to stunting among children under five specifically in four Northern African countries, Algeria, Egypt, Sudan, and Tunisia

Variable	Algeria 2018-19		Egypt 2014		Sudan 2014		Tunisia 2017-18	
	N	%	N	%	N	%	N	%
	Household factors							
**Place of residence**								
Urban	9458	57.6	4411	87.3	3568	27.8	2288	63.3
Rural	6961	42.4	642*	12.7	9273	72.2	1328	36.7
**Family size**								
2-4 members	4366	26.6	1997	39.5	2269	17.7	1654	45.8
5-10 members	10966	66.8	2879	57.0	9295	72.4	1936	53.5
>10 members	1086	6.6	177	3.5	1278	10.0	25	0.7
**Number of children < 5**								
1	9245	56.3	2127	42.1	3932	30.62	2061	57.0
2 or more	7174	43.7	2926	57.9	8910	69.38	1554	43.0
**Type of cooking fuels**								
Clean	16327	99.4	4995	98.8	4981	38.8	3604	99.8
Unclean	92	0.6	59	1.2	7861	61.2	8	0.2
**Household wealth quantile**								
Poorest	2962	18.0	1290	25.5	2453	19.1	817	22.6
Poorer	3353	20.4	1121	22.2	2026	15.8	744	20.6
Middle	3208	19.5	1090	21.6	2221	17.3	694	19.2
Fourth	3307	20.1	863	17.1	2842	22.1	665	18.4
Richest	3589	21.9	689	13.6	3299	25.7	696	19.3
**Sources of drinking water**								
Improved	11694	71.2	4897	96.9	10006	78.1	1921	53.1
Unimproved	4721	28.8	156	3.1	2813	21.9	1695	46.9
**Type of toilet facility**								
Improved	10058	61.3	4844	96.0	4718	36.8	1467	40.6
Unimproved	6358	38.7	201	4.0	8094	63.2	2148	59.4
**Literacy**								
Yes	3277	23.1	288	17.2	3398	35.6	554	16.6
No	10935	76.9	1391	82.9	6142	64.4	2782	83.4
	Care giver's related factors							
**Maternal level of education**								
Secondary and higher	6626	46.5	2719	53.8	2903	22.6	2407	66.6
Primary	5157	36.2	1105	21.9	4529	35.3	919	25.4
None	2455	17.2	1230	24.3	5394	42.1	289	8.0
**Maternal age (years)**								
15-19 years	1007	6.3	127	2.5	485	3.9	4**	0.1
20-34 years	9179	57.2	4082	80.9	8481	67.8	2060	57.7
35-49years	5858	36.5	836	16.6	3545	28.3	1509	42.2
**Paternal age (years)**								
18-34	11	0.1	2561	51.4	3294	27.1	739	22.6
35-44	10566	77.3	1867	37.5	5002	41.2	1851	56.7
45+	3096	22.6	557	11.2	3852	31.7	675	20.7

**Maternal marital status**								
Married	13668	85.2	4986	98.8	12153	97.2	3265	91.3
Unmarried	2376	14.8	59	1.2	357	2.9	310	8.7
**Maternal age at marriage**								
≤ 18 years	748	5.3	1485	29.44	6343	50.9	96	2.9
>18 years	13470	94.7	3560	77.56	6124	49.1	3211	97.1
**Maternal body mass index (BMI)**								
18.5-24.9	2879	17.6	1107	21.9	580	4.5	801	22.2
<18.5	13319	81.4	3868	76.6	12140	94.8	2771	76.7
25+	157	1.0	79	1.6	92	0.7	43	1.2
**Listening to the radio**								
No	13203	82.3	4197	83.2	8465	66.0	1840	51.5
Yes	2835	17.7	848	16.8	4364	34.0	1734	48.5
**Watching TV**								
No	1056	6.6	118	2.3	8002	62.3	83	2.3
Yes	14986	93.4	4926	97.7	4838	37.7	3490	97.7
	Child related factors							
**Child's sex**								
Boy	8464	51.6	2504	49.55	6534	50.9	1868	51.7
Girl	7955	48.5	2550	50.45	6308	49.1	1747	48.3
**Child's age (months)**								
0-5 m	1518	9.2	578	11.4	1220	9.5	310	8.6
6-11 m	1564	9.5	552	10.9	1360	10.6	317	8.8
12-17 m	1750	10.7	567	11.2	1397	10.9	334	9.2
18-23	1561	9.5	519	10.3	1105	8.6	356	9.8
24-29	1554	9.5	550	10.9	1351	10.5	325	9.0
30-35	1722	10.5	456	9.0	1079	8.4	376	10.4
36-41	1826	11.1	519	10.3	1588	12.4	353	9.8
42-47	1612	9.8	446	8.8	1386	10.8	397	11.0
48-53	1587	9.7	463	9.2	1312	10.2	455	12.6
54-59	1725	10.5	403	8.0	1044	8.1	393	10.9
**Child's birth order**								
1	6705	62.4	2163	42.9	4408	35.1	1301	67.6
>2	4037	37.6	2879	57.1	8151	64.9	625	32.5
	Health facility access factors							
**Place of childbirth**								
Health facility	5239	98.7	1913	86.6	1467	29.5	1199	99.7
Home	68	1.3	297	13.5	3497	70.5	3***	0.3
**Number of antenatal visits**								
8+	637	3.9	2313	45.8	546	4.3	408	11.3
4-7 visits	4655	28.4	1725	34.1	3903	30.4	1029	28.5
1-3 visits	2181	13.3	499	9.9	2527	19.7	221	6.1
None	8946	54.5	517	10.2	5865	45.7	1957	54.1
**Delivery assistants**								
Health professionals	7622	46.4	4498	89	6838	53.3	1731	47.9

Non-Health professionals	8797	53.6	556	11	6004	46.8	1885	52.1
**Type of delivery**								
Non-caesarean	3883	74.2	920	48.1	985	66.0	669	55.8
Caesarean	1349	25.8	993	51.9	506	34.0	529	44.2
**Postnatal check up**								
0-2 days	1051	6.4	1137	22.5	914	7.1	265	7.3
After 2	417	2.5	83	1.6	54	0.4	278	7.7
No	14951	91.1	3833	75.9	11873	92.5	3072	85.0
	Dietary intake							
**Dietary diversity**								
5+ food groups	2224	34.8	759	34.3	863	17.0	731	55.5
<5 food groups	4168	65.2	1456	65.7	4219	83.0	587	44.5
**Early Initiation of breastfeeding**								
After 1 hour	4093	64.0	1162	52.5	1141	22.5	849	64.5
Within 1 hour	2299	36.0	1054	47.6	3941	77.6	468	35.5
**Duration of breastfeeding**								
Up to 12 months	2500	39.1	1185	53.5	2690	52.9	556	42.2
>12 months	3892	60.9	1031	46.5	2392	47.1	762	57.8
	Child health							
**Perceived child size at birth**								
Average	3510	66.8	1517	68.8	2586	52.7	839	69.8
Small	809	15.4	561	25.5	1691	34.5	153	12.8
Large	934	17.8	126	5.7	627	12.8	209	17.4
**Diarrhea in the last two weeks**								
No	15295	93.4	4083	80.8	8904	69.9	3319	92.1
Yes	1085	6.6	971	19.2	3839	30.1	286	7.9
**Cough in the last two weeks**								
No	12460	76.0	3317	65.7	8763	68.5	2536	70.2
Yes	3946	24.1	1736	34.4	4037	31.5	1077	29.8
**Fever**								
No	13762	84.0	4058	80.3	2672	20.8	2867	79.4
Yes	2628	16.0	995	19.7	10170	79.2	742	20.6
**Any infection**								
No	11245	68.5	2686	53.2	6509	50.7	2229	61.6
Yes	5174	31.5	2367	46.8	6332	49.3	1387	38.4

* = Data from upper Egypt predominantly rural areas

** = small number of respondents who's their age less than 19 years old

*** = Only 3 mothers give birth at home in Tunisian survey

Table S2. Prevalence and 95% confidence intervals (CIs) of stunting among children (0-23, 24-59 and 0-59 months) in four Northern African countries, Algeria 2018-19 (N= 16419), Egypt 2014 (N=5053), Sudan 2014 (N= 12841), and Tunisia 2017-18 (N= 3615)

	**Algeria 2019**			**Egypt 2014**			**Sudan 2014**			**Tunisia 2018**		
	**0-23 months**	**24-59 months**	**0-59 months**	**0-23 months**	**24-59 months**	**0-59 months**	**0-23 months**	**24-59 months**	**0-59 months**	**0-23 months**	**24-59 months**	**0-59 months**
**Variable**	**Pr (95%CI)**	**Pr (95%CI)**	**Pr (95%CI)**	**Pr (95%CI)**	**Pr (95%CI)**	**Pr (95%CI)**	**Pr (95%CI)**	**Pr (95%CI)**	**Pr (95%CI)**	**Pr (95%CI)**	**Pr (95%CI)**	**Pr (95%CI)**
Household factors												
**Place of residence**												
Urban	8.4 [7.2,9.8]	9.9 [8.5,11.5]	9.3 [8.3, 10.5]	23.1 [20.1,25.7]	20.3 [18.0,22.8]	21.5 [19.7,23.4]	19.6 [16.7,22.8]	28.9 [25.5,32.5]	25.3 [22.6,28.1]	7.3 [5.5,9.6]	7.5 [6.0,9.3]	7.4 [6.3,8.8]
Rural	9.3 [7.3,11.7]	10.8 [9.3,12.6]	10.2 [8.9, 11.7]	16.2 [13.0, 19.9]	20.1 [16.8, 23.8]	18.4 [15.8, 21.2]	27.8 [25.5,30.2]	43.2 [40.3,46.1]	37 [34.8,39.3]	10.6 [7.5,14.6]	8.7 [5.9,12.6]	9.4 [7.0,12.6]
**Family size**												
2-4 members	9.1 [7.3,11.3]	10.0 [8.5,11.8]	9.6 [8.5, 11.0]	20.1[17.0, 23.7]	20.4 [17.5, 23.6]	20.3 [17.9, 22.9]	24.1 [20.8,27.7]	38.4 [34.6,42.3]	31.4 [28.9,34.1]	8.2 [6.1,10.9]	7.6 [5.9,9.7]	7.8 [6.4,9.5]
5-10 members	9.1 [7.8, 10.6]	10.6 [9.3, 12.0]	10.0 [9.0, 11.1]	24.0 [21.0, 27.2]	20.0 [17.4, 22.9]	21.5 [19.5, 23.7]	25.3 [23.1,27.5]	39.6 [37.1,42.3]	34.3 [32.3,36.3]	8.9 [6.6,11.8]	8.3 [6.3,10.8]	8.5 [6.8,10.5]
>10 members	5.1 [2.8, 9.0]	8.6 [5.4, 13.3]	6.9 [4.8, 9.9]	25.3 [14.1, 41.0]	23.1 [14.5, 34.9]	23.8 [15.9, 34.0]	30.2 [23.1,38.4]	36.6 [31.6,41.8]	33.9 [30.2,37.9]	7.7 [1.0,40.9]	5.7 [0.7,32.8]	6.4 [1.5,23.5]
**Number of children < 5**												
1	8.8 [7.3,10.5]	11.6 [10.1,13.3]	10.6 [9.4,11.8]	21 [18.1,24.3]	19.4 [16.6,22.6]	20.2 [18.1,22.4]	25.6 [22.8,28.5]	40.6 [37.2,44.0]	34.7 [32.3,37.2]	7.1 5.1,9.7]	7.7 [6.1,9.7]	7.5 [6.2,9.1]
2 or more	8.8 [7.5,10.3]	8.5 [7.4,9.8]	8.6 [7.7,9.7]	23.2 [20.2,26.5]	20.8 [18.3,23.6]	21.8 [19.7,24.1]	25.5 [23.3,27.8]	38.5 [36.0,41.0]	33.3 [31.4,35.3]	10.3 [7.8,13.6]	8.3 [6.4,10.7]	9.1 [7.3,11.2]
**Type of cooking fuels**												
Clean	8.8 [7.0, 10.1]	10.3 [9.2, 11.4]	9.7 [8.9, 10.6]	22.2 [19.9, 24.5]	20.3 [18.3, 22.6]	21.1 [19.5, 22.8]	21.1 [18.0,24.5]	34.9 [30.8,39.4]	29.3 [26.5,32.4]	8.5 [6.8,10.5]	7.9 [6.5,9.6]	8.1 [6.9,9.5]
Unclean	2.0 [0.3,12.2]	17.4 [8.7, 31.7]	13.1 [6.4,24.9]	25.7 [12.1, 46.6]	14.0 [4.4, 36.6]	19.8 [10.7, 33.8]	28.5 [26.2,30.9]	41.7 [39.0,44.6]	36.6 [34.3,38.9]	0.0	22.6 [3.2,71.9]	16.4 [3.3,53.1]
**Household wealth quantile**												
Richest	6.8 [5.1, 9.2]	13.7 [11.4,16.3]	14.1 [12.1,6.4]	20.6 [16.5,25.5]	14.3 [10.8, 18.6]	17.1 [14.2, 20.5]	18 [14.4,22.1]	28.3 [24.2,32.7]	24.2 [21.1,27.5]	8.2 [5.3,12.5]	7.5 [5.3,10.4]	7.8 [5.9,10.1]
Fourth	7.2 [5.5, 9.3]	10.5 [8.6, 12.7]	9.7 [8.2, 11.5]	18.7 [14.4,24.1]	19.4 [15.2, 24.4]	19.1 [15.7, 23.0]	26.3 [23.2,29.6]	43.8 [39.7,48.1]	36.6 [33.9,39.4]	8.2 [5.3,12.5]	7 [4.8,10.1]	7.4 [5.6,9.8]
Middle	7.5 [5.7, 9.8]	7.5 [5.9, 9.4]	7.5 [6.2, 9.0]	23.9 [19.2,29.2]	22.2 [17.7, 27.6]	23.0 [19.6, 26.8]	31.8 [28.5,35.4]	49 [44.9,53.1]	41.9 [38.8,45.0]	4.4 [2.4,7.8]	8.1 [5.6,11.6]	6.8 [5.0,9.2]
Poorer	8.7 [6.7, 11.3]	9.7 [7.9, 12.0]	8.7 [7.4, 10.3]	23.7 [19.9,28.1]	19.5 [15.6, 24.1]	21.2 [18.3, 24.5]	27.9 [24.1,32.0]	41.7 [37.7,45.8]	36.2 [32.9,39.6]	11.9 [8.1,17.1]	7.8 [4.9,12.3]	9.2 [6.5,12.9]
Poorest	14.8 [11.2,19.3]	10.3 [8.0, 13.3]	9.0 [7.3, 11.0]	22.8 [18.7,27.6]	23.0 [19.2, 27.2]	22.9 [20.2, 25.9]	27 [22.5,32.0]	37.8 [33.0,43.0]	34 [29.5,38.8]	10.2 [7.0,12.6]	9.6 [5.7,15.6]	9.8 [6.6,14.2]
**Sources of drinking water**												

Improved	8.9 [7.6, 10.3]	10.5 [9.2, 11.9]	9.8 [8.9, 10.9]	22.1 [19.9, 24.5]	20.4 [18.3, 22.7]	21.2 [19.6, 22.9]	24.4 [22.3,26.6]	38.2 [35.4,41.0]	32.6 [30.5,34.8]	9.3 [7.1,12.2]	6.3 [4.9,8.0]	7.4 [6.1,9.0]
Unimproved	8.5 [6.8, 10.7]	9.9 [8.2, 12.0]	9.4 [8.0, 11.0]	24.5 [14.0, 39.4]	16.0 [10.7, 23.2]	19.4 [13.1, 27.7]	29.9 [26.6,33.5]	42.8 [39.1,46.5]	38 [35.1,41.0]	7.6 [5.6,10.3]	9.8 [7.4,12.8]	9 [7.2,11.2]
**Type of toilet facility**												
Improved	9.1[7.8, 10.5]	10.7 [9.4, 12.3]	10.1 [9.1, 11.2]	22.0 [19.8, 24.4]	20.6 [18.5, 22.8]	21.2 [19.6, 22.9]	22 [18.9,25.5]	33 [29.5,36.7]	28.6 [25.8,31.6]	8.7 [6.2,12.0]	6.3 [4.7,8.4]	7.2 [5.7,9.1]
Unimproved	8.3 [6.8, 10.3]	9.6 [8.2, 11.3]	9.1 [7.9, 10.5]	27.9 [18.6, 39.6]	13.0 [8.4, 19.5]	19.8 [15.1, 25.6]	27.6 [25.4,29.9]	42.9 [40.0,45.7]	36.9 [34.6,39.1]	8.5 [6.5,11.0]	9 [7.1,11.5]	8.8 [7.3,10.7]
**Literacy**												
Yes	8.6 [6.5, 11.2]	10.3 [8.3, 12.6]	9.7 [8.1, 11.5]	30.0 [20.4,41.8]	19.7 [13.2,28.4]	23.7 [17.6,31.0]	23.8 [20.6,27.3]	41.7 [38.4,45.1]	34.3 [31.8,36.9]	8.4 [5.2,13.4]	5.7 [3.6,8.9]	6.8 [5.0,9.2]
No	8.2 [7.0, 9.6]	9.5 [8.4, 10.8]	9.0 [8.1, 9.9]	22.0 [17.6,27.0]	22.1 [18.5,26.2]	22.1 [19.2,25.2]	29.2 [26.4,32.0]	44 [40.9,47.2]	38.3 [35.8,40.9]	8.1 [6.4,10.3]	8 [6.5,9.7]	8 [6.8,9.5]
Care giver's related factors												
**Maternal level of education**												
Secondary and higher	7.7 [6.1, 9.6]	9.1 [7.5, 11.1]	8.5 [7.3, 9.9]	20.5 [17.6,23.9]	20.3 [17.7,23.2]	20.4 [18.2,22.8]	21 [17.9,24.6]	26 [22.4,29.9]	23.9 [21.1,26.8]	7.7 [6.0,9.9]	7.3 [5.9,9.0]	7.5 [6.3,8.8]
Primary	8.8 [6.9, 11.2]	9.4 [7.9, 11.1]	9.2 [7.9, 10.6]	26.0 [21.8,30.8]	18.2 [14.3,22.8]	21.8 [19.0,25.0]	24.5 [22.0,27.3]	40.3 [37.1,43.6]	33.8 [31.4,36.3]	9 [5.5,14.5]	8.4 [6.1,11.4]	8.6 [6.4,11.4]
None	7.4 [5.4, 10.1]	10.9 [8.6, 13.7]	9.6 [7.9, 11.7]	22.3 [17.9,27.4]	21.8 [18.0,26.2]	22 [19.1,25.2]	29.3 [26.5,32.2]	44.8 [41.6,48.0]	39.1 [36.6,41.7]	14.8 [7.9,25.9]	11.4 [6.3,19.8]	12.6 [8.2,18.7]
**Maternal age (years)**												
15-19 years	9.2 [5.9, 14.0]	11.5 [8.9, 14.9]	10.8 [8.7, 13.5]	23.6 [14.8,35.5]	30.5 [10.8,61.5]	24.4 [16.1,35.2]	29.9 [23.3,37.4]	40.3 [31.3,50.0]	32.9 [27.7,38.6]			
20-34 years	8.9 [7.5, 10.5]	10.5 [9.1, 12.0]	9.8 [8.8, 10.9]	22 [19.8,24.5]	19.8 [17.6,22.2]	20.8 [19.1,22.6]	24.8 [22.8,27.0]	40.4 [37.7,43.1]	33.9 [32.0,35.8]			
15-34 years*										7.9 [6.1, 10.2]	7.5 [5.9,9.5]	7.7 [6.4, 9.3]
35-49years	8.9 [7.4, 10.7]	10.0 [8.7, 11.4]	9.6 [8.6, 10.8]	22.5 [17.5,28.5]	22.2 [18.4,26.6]	22.3 [19.2,25.8]	26.7 [23.4,30.3]	37.6 [33.7,41.7]	34.1 [30.8,37.5]	10 [7.0,13.9]	8.7 [6.6,11.2]	9 [7.3, 11.2]
**Paternal age (years)**												
18-34	21.1[2.8,71.2]	73.6 [19.8,96.9]	41.9 [18.0,70.3]	22.7 [19.8,25.9]	23.1 [20.2,26.3]	22.9 [20.7,25.2]	25.5 [22.6,28.6]	43.3 [39.7,46.8]	34.7 [32.2,37.3]	5.8 [3.8,8.9]	6.7 [4.0,11.0]	6.3 [4.3,9.0]
35-44	9.3 [8.0, 10.8]	10.1 [9.0, 11.3]	9.8 [8.8, 10.7]	22 [18.6,25.8]	16.9 [14.4,19.8]	18.9 [16.7,21.3]	24.1 [21.5,27.0]	37.8 [34.8,40.9]	32.2 [30.0,34.6]	9.8 [7.6,12.7]	8.9 [7.2,10.9]	9.2 [7.7,11.0]
45+	7.8 [5.9, 10.2]	10.0 [8.5, 11.8]	9.3 [8.0, 10.8]	18.9 [13.1,26.5]	21.6 [17.0,27.0]	20.6 [17.0,24.8]	28.3 [25.0,31.8]	39.6 [36.4,43.0]	35.7 [33.0,38.6]	8 [5.0,12.5]	6.4 [4.4,9.2]	6.9 [5.1,9.1]
**Maternal marital status**												
Married	9.0 [7.9,10.4]	10.1 [9.1, 11.2]	9.7 [8.9, 10.6]	22.1 [19.9,24.5]	20.4 [18.3,22.6]	21.1 [19.5,22.8]	25.7 [23.8,27.6]	39.7 [37.3,42.2]	34 [32.2,35.9]	8.3 [6.7,10.3]	7.9 [6.5,9.5]	8.1 [6.9,9.4]
Unmarried	8.0 [5.8,11.0]	11.7 [9.2, 14.6]	10.4 [8.6, 12.6]	33 [11.4,65.4]	18.3 [8.4,35.6]	21.7 [11.4,37.3]	20.2 [12.7,30.5]	32.7 [26.6,39.5]	29.2 [24.1,34.8]	10.8 [5.5,20.2]	9.2 [5.5,14.9]	9.7 [6.4,14.3]

**Maternal age at marriage**												
≤ 18 years	9.6 [6.4,14.1]	10.2 [7.4, 13.8]	9.9 [7.8, 12.7]	20.3 [18.8,24.0]	18.1 [15.0,21.6]	19 [16.8,21.5]	27.5 [25.0,30.2]	42.7 [39.9,45.4]	36.6 [34.4,38.9]	6.4 [1.6,22.8]	7.3 [3.1,16.3]	7 [3.4,13.8]
>18 years	8.9 [7.7,10.3]	10.0 [9.0, 11.1]	9.6 [8.7, 10.4]	22.9 [20.1,26.0]	21.3 [18.9,24.0]	22 [20.1,24.1]	23.5 [21.1,26.0]	36.3 [33.3,39.4]	31.1 [29.0,33.3]	8.4 [6.8,10.4]	8 [6.6,9.7]	8.1 [6.9,9.5]
**Maternal body mass index (BMI)**												
<18.5	6.3 [5.2, 7.5]	7.6 [6.7, 8.6]	7.1 [6.4, 7.9]	18.3 [12.2,40.3]	16.7 [14.6,19.0]	17.3 [15.7,19.1]	24.8 [22.9,26.7]	38.2 [35.8,40.6]	33 [31.2,34.9]	5.9 [4.3,8.0]	4.8 [3.8,6.1]	5.1 [4.2,6.2]
18.5-24.9	15.0 [12.5,17.9]	27.5 [23.5,31.9]	20.4 [17.9,23.1]	31 [26.1,36.3]	36.4 [31.6,41.6]	33.3 [29.6,37.2]	37.2 [30.6,44.3]	71 [60.1,79.9]	51.3 [44.4,58.1]	12.7 [9.5,16.9]	21.4 [16.3,27.4]	16.8 [13.8,20.4]
25+	37.3 [22.1,55.4]	40.0 [27.0,54.7]	39.0 [28.3,50.8]	33.8 [18.7,53.1]	40.1 [25.9,56.2]	36.5 [25.3,49.4]	18.1 [8.0,35.9]	19.8 [10.3,34.7]	19.2 [11.7,29.9]	38.9 [16.0,68.0]	43.9 [26.5,62.9]	41.7 [26.9,58.2]
**Listening to the radio**												
No	9.3 [8.0,10.8]	10.6 [9.5, 11.9]	10.1 [9.2, 11.1]	22.6 [20.3,25.2]	20.8 [18.5,23.3]	21.6 [19.9,23.5]	26.4 [24.2,28.6]	41.1 [38.6,44.1]	35.5 [33.5,37.6]	8.7 [6.7,11.2]	9.1 [6.8,12.0]	8.9 [7.2,11.0]
Yes	7.3 [5.7. 9.4]	9.0 [6.9, 11.5]	8.3 [6.9, 10.0	19.9 [15.7,24.8]	17.9 [14.1,22.5]	18.8 [15.8,22.1]	24 [21.1,27.2]	34.8 [31.9,37.9]	30.5 [28.2,32.8]	8.3 [6.2,11.1]	7 [5.4,8.9]	7.4 [6.1,9.0]
**Watching TV**												
No	13.5 [9.8,18.4]	13.1 [10.3,16.5]	13.2 [10.9,16.1]	13.3 [4.7,32.2]	22.8 [12.7,37.5]	18.8 [11.1,30.1]	28.1 [25.9,30.5]	43.6 [40.7,46.5]	37.5 [35.2,39.9]	13.1 [5.0,30.0]	13.1 [6.3,25.1]	13.1 [6.8,23.7]
Yes	8.6 [7.4, 9.8]	10.2 [9.1,11.4]	9.5 [8.7, 10.4]	22.4 [20.2,24.8]	20.3 [18.2,22.5]	21.2 [19.6, 22.9]	21.3 [18.2,24.8]	31.6 [28.5,34.9]	27.5 [25.2,30.0]	8.4 [6.7,10.4]	7.9 [6.4,9.7]	8.1 [6.8,9.5]
Child related factors												
**Child's sex**												
Boy	11.4 [9.7,13.3]	11.5 [10.1,13.1]	11.5 [10.3,12.7]	23.7 [20.6,27.0]	20.4 [17.5,23.7]	21.9 [19.7,24.2]	28.9 [26.5,31.5]	40 [37.5,42.6]	35.7 [33.6,37.8]	8.2 [6.3,10.7]	7.3 [5.6,9.4]	7.6 [6.3,9.2]
Girl	6.0 [4.9,7.3]	9.0 [7.7,10.5]	7.9 [6.9,8.9]	20.7 [17.7,24.2]	20.1 [17.5,22.9]	20.4 [18.3,22.6]	22 [19.6,24.6]	38.2 [35.3,41.2]	31.8 [29.8,33.8]	8.9 [6.4,12.2]	8.6 [6.7,11.1]	8.7 [7.0,10.9]
**Child's age (months)**												
0-5	9.4 [7.2,12.0]			19.5 [15.7,24.0]			11 [8.7,13.8]			11.3 [7.7,16.2]		
6-11 m	6.5 [5.0,8.4]			20.1 [16.3,24.6]			17.4 [14.9,20.2]			7 [4.0,11.8]		
12-17m	8.2 [6.4,10.3]			22.6 [18.6,27.2]			32.9 [29.6,36.4]			5.5 [3.4,8.8]		
18-23	11.2 [9.1,13.7]			27 [22.0,32.6]			42.2 [38.1,46.5]			10.3 [7.3,14.5]		
24-29		10.6 [8.2, 13.5]			28.7 [24.2,33.7]			43 [39.4,46.6]			10.4 [6.9,15.4]	
30-35		12.4 [10.1,15.0]			34.4 [29.2,40.1]			46.2 [41.8,50.7]			9.5 [6.7,13.2]	
36-41		8.9 [7.1, 11.2]			15 [11.6,19.2]			34.9 [31.0,39.0]			6 [3.5,9.9]	

42-47		10.7 [8.7, 13.2]			17.5 [13.7,22.1]			45.7 [41.8,49.7]			10.2 [7.3,14.0]	
48-53		7.8 [5.8, 10.3]			14.1 [10.4,18.8]			33.4 [28.8,38.2]			5.4 [3.3,8.7]	
54-59		10.3 [9.3, 11.5]			9.6 [6.7 ,13.8]			39.1[36.8,41.6]			8 [4.6,10.2]	
0-23 m			8.8 [7.7, 10.1]			22.2 [20.0,24.6]			25.5 [23.7,27.5]			8.5 [6.9,10.6]
24-59 m			10.3 [9.3, 11.5]			20.3 [18.2,22.5]			39.1 [36.8,41.6]			8 [6.5,9.7]
**Child's birth order**												
1	8.9 [7.2,11.0]	9.3 [7.9,10.8]	9.1 [8.0, 10.4]	23 [19.8,26.7]	19.4 [16.5,22.8]	21.3 [18.8,24.0]	25.9 [23.4,28.7]	40.1 [36.8,43.4]	34.2 [31.8,36.7]	6.7 [4.4,10.0]	7.3 [5.2,10.1]	7.1 [5.4,9.4]
>2	8.2 [6.4, 10.4]	12.2 [10.2,14.5]	10.8 [9.3, 12.5]	21.4 [18.5,24.6]	20.9 [18.3,23.7]	21.1 [18.8,24.0]	26.2 [23.8,28.9]	38.7 [36.2,41.2]	34.2 [32.2,36.1]	11.9 [7.2,19.1]	7.4 [5.0,10.8]	8.9 [6.5,12.0]
Health facility access factors												
**Place of childbirth**												
Health facility	9.2 [8.0, 10.6]			22.3 [20.0,24.9]	21.7 [18.3,25.5]		22.3 [18.7,26.3]			8.5 [6.8,10.5]		
Home	10.1 [5.2,18.8]			21.5 [16.5,27.5]	23 [20.1,26.1]		27.2 [25.1,29.3]			0.0		
**Number of antenatal visits**												
8+	8.0 [5.6,11.4]	5.6 [2.7,11.4]	7.4 [5.3,10.2]	21.8 [19.1,24.9]	18 [15.3,21.0]	19.9 [17.9,22.1]	22 [16.3,29.1]	29.6 [22.1,38.3]	25 [19.9,30.8]	8.9 [5.9,13.1]	3.8 [1.4,10.2]	7.5 [5.0,11.1]
4-7 visits	8.7 [7.3,10.5]	8.8 [7.1,10.8]	8.7 [7.5,10.2]	22.9 [19.2,27.1]	21.3 [17.8,25.4]	22 [19.2,25.0]	23.9 [21.2,26.7]	35.1 [31.6,38.7]	28.6 [26.2,31.1]	8.2 [6.1,11.0]	8.8 [5.9,13.1]	8.4 [6.4,11.0]
1-3 visits	10.0 [8.3,12.0]	8.1 [5.9,10.9]	9.3 [8.0,10.9]	22 [16.2,29.3]	22 [16.3,29.0]	22 [18.3,26.3]	26 [23.2,29.0]	43.9 [39.1,48.8]	33.9 [31.0,37.1]	6.7 [3.6,12.4]	4.3 [1.6,11.0]	5.8 [3.3,10.2]
None	7.8 [6.0,10.1]	10.9 [9.6,12.3]	10.5 [9.3,11.7]	21.7 [14.8,30.6]	23.2 [18.7,28.5]	22.7 [18.4,27.7]	29.4 [26.0,33.1]	39.8 [37.2,42.5]	37.9 [35.5,40.4]	10.8 [6.6,17.1]	8.2 [6.6,10.2]	8.4 [6.9,10.3]
**Delivery assistants**												
Health professionals	9.2 [8.0, 10.5]	8.6 [7.2, 10.2]	9.0 [8.0, 10.1]	22.4 [20.1,24.8]	20.4 [18.3,22.8]	21.3 [19.6,23.1]	24.8 [22.8,26.9]	37.8 [34.6,41.0]	30.3 [28.3,32.3]	8.5 [6.9,10.5]	7.2 [5.1,10.0]	8.1 [6.6,9.9]
Non-Health professionals	7.0 [5.2, 9.4]	10.8 [9.6,12.2]	10.3 [9.2, 11.6]	20.4 [14.6,27.8]	19.2 [15.0,24.2]	19.6 [16.2,23.5]	28.1 [24.7,31.8]	40 [37.1,42.9]	37.7 [35.1,40.4]	8.8 [4.4,16.9]	8.2 [6.6,10.1]	8.2 [6.7,10.1]
**Type of delivery**												
Non -caesarean	9.5 [8.2, 11.1]			21.7 [18.3,25.5]			20.5 [16.6,25.0]			9.2 [7.0,12.0]		
Caesarean	8.5 [6.6, 10.7]			23 [20.1,26.1]			25.4 [20.6,30.8]			7.7 [5.6,10.5]		
**Postnatal check up**												
0-2 days	8.1 [5.6,11.6]	9.2 [6.0,13.8]	8.4 [6.3,11.2]	19.8 [16.4,23.6]	21.7 [17.7,26.3]	20.8 [18.0,24.0]	28.4 [23.7,33.7]	34.4 [27.4,42.1]	31.2 [26.7,36.1]	10.8 [6.9,16.6]	4.1 [1.3,11.9]	8.6 [5.6,13.0]

After 2	13.5 [9.0,19.7]	8.3 [3.8,17.3]	11.9 [8.1,17.0]	27.2 [16.7,41.0]	15.6 [6.9,31.7]	21.6 [15.6,29.2]	29.6 [13.9,52.2]	24.8 [7.9,56.0]	27.5 [13.8,47.4]	6.9 [3.7,12.5]	5.3 [2.0,13.5]	6.5 [3.9,10.6]
No	8.6 [7.4,10.0]	10.4 [9.3,11.6]	9.7 [8.9,10.7]	22.8 [20.3,25.6]	19.9 [17.7,22.3]	21.2 [19.4,23.1]	25.2 [23.3,27.2]	39.5 [37.0,41.9]	34 [32.1,35.9]	8.5 [6.5,11.0]	8.2 [6.7,10.0]	8.3 [7.0,9.8]
Dietary intake												
**Dietary diversity**												
5+ food groups	7.9 [6.4, 9.7]			24.4 [20.8,28.3]			27 [22.6,32.0]			7.2 [5.3,9.6]		
<5 food groups	9.2 [7.9,10.8]			21.1 [18.5,23.9]			25.2 [23.4,27.1]			10.2 [7.5,13.8]		
**Early Initiation of breastfeeding**												
After 1 hour	8.6 [7.4, 9.9]			21.3 [18.5,24.3]			26.5 [22.4,31.0]			8.1 [6.1,10.6]		
Within 1 hour	9.2 [7.5, 11.1]			23.2 [20.2,26.6]			25.2 [22.4,31.0]			9.4 [6.9,12.7]		
**Duration of breastfeeding**												
Up to 12 months	8.3 [6.8, 10.1]			20.1 [17.2,23.3]			15 [13.2,17.0]			8.2 [5.9,11.4]		
>12 months	9.1 [7.8, 10.6]			24.6 [21.2,28.4]			37.4 [34.3,40.5]			8.8 [6.8,11.3]		
Child health												
**Perceived baby size**												
Average	9.0 [7.6, 10.7]			20.4 [18.1,22.9]			23.2 [20.7,25.8]			8.6 [6.6,11.0]		
Small	12.6 [9.9,15.8]			28.2 [23.8,33.2]			30.2 [27.1,33.3]			15.4 [9.7,23.5]		
Large	6.5 [4.6, 9.0]			18.1 [11.2,27.8]			23.6 [19.8,27.7]			3.1 [1.5,6.4]		
**Diarrhea**												
No	9.1 [7.9, 10.4]	10.4 [9.4, 11.6]	9.9 [9.1, 10.8]	22.5 [19.8,25.5]	20 [17.9,22.2]	21 [19.2,22.8]	25.2 [23.1,27.5]	37.2 [34.6,39.8]	32.9 [30.8,35.0]	8.2 [6.6,10.2]	8 [6.6,9.8]	8.1 [6.9,9.5]
Yes	6.0 [3.2,10.7]	7.6 [4.6, 12.1]	6.6 [4.5, 9.7]	21.4 [17.9,25.3]	22.6 [17.8,28.3]	21.8 [18.9,24.9]	26 [23.1,29.2]	45.2 [41.4,49.1]	36.1 [33.7,38.5]	10.9 [6.0,19.1]	6.5 [3.2,12.9]	9 [5.5,14.3]
**Cough**												
No	8.5 [7.2, 9.9]	10.1 [9.0, 11.4]	9.5 [8.6, 10.4]	23.1 [20.3,26.2]	20.7 [18.3,23.4]	21.8 [19.9,23.8]	24.6 [22.6,26.8]	39 [36.1,41.9]	33.1 [30.8,35.4]	8.5 [6.6,10.9]	8.9 [7.1,11.0]	8.7 [7.3,10.5]
Yes	9.7 [7.7,12.2]	10.9 [8.8, 13.4]	10.4 [8.8, 12.3]	20.4 [17.0,24.2]	19.4 [16.3,22.8]	19.8 [17.6,22.2]	27.9 [24.5,31.4]	39.6 [36.9,42.4]	35.3 [33.4,37.4]	8.6 [5.8,12.8]	5.7 [4.0,7.9]	6.8 [5.2,8.8]
**Fever**												
No	8.6 [7.4,10.0]	10.3 [9.2, 11.5]	9.7 [8.8, 10.6]	22.7 [20.2,25.4]	20.6 [18.3,23.0]	21.5 [19.7,23.3]	26.2 [22.6,30.2]	36.2 [32.8,39.8]	32.7 [29.6,35.9]	8.5 [6.7,10.7]	8.4 [6.8,10.2]	8.4 [7.1,10.0]
Yes	9.5 [7.0, 12.8]	10.5 [8.2, 13.3]	10.1 [8.3,12.2]	20.4 [16.2,25.5]	18.9 [15.2,23.2]	19.6 [16.7,22.9]	25.4 [23.3,27.5]	40 [37.3,42.7]	34 [32.0,36.1]	8.3 [5.1,13.2]	6 [3.8,9.2]	7 [5.0,9.6]
**Any infection**												

No	8.8 [7.4,10.3]	10.2 [9.0, 11.5]	9.6 [8.7, 10.7]	23.7 [20.6,27.2]	20.5 [17.9,23.3]	21.8 [19.7,23.9]	24.9 [22.4,27.5]	37.7 [34.6,40.9]	32.8 [30.3,35.5]	8.8 [6.7,11.4]	9.2 [7.4,11.3]	9 [7.6,10.8]
Yes	8.8 [7.1,11.0]	10.6 [8.8,12.7]	9.9 [8.4, 11.5]	20.8 [18.0,23.9]	20 [17.1,23.1]	20.4 [18.3,22.6]	26.1 [23.7,28.7]	40.7 [38.0,43.4]	34.7 [32.8,36.6]	8.2 [5.9,11.3]	5.7 [4.2,7.8]	6.8 [5.3,8.6]

Table S3. Multivariable analysis of stunting among children (0-23 months) in four Northern African countries, Algeria 2018-19 (N= 16419), Egypt 2014 (N=5053), Sudan 2014 (N= 12841), and Tunisia 2017-18 (N= 3615)

	**Algeria 2018-19**				**Egypt 2014**				**Sudan 2014**				**Tunisia 2017-18**			
Variable	**OR (95%CI)**	**P-value**	**AOR (95%CI)**	**P-value**	**OR (95%CI)**	**P-value**	**AOR (95%CI)**	**P-value**	**OR (95%CI)**	**P-value**	**AOR (95%CI)**	**P-value**	**OR (95%CI)**	**P-value**	**AOR (95%CI)**	**P-value**
Household factors																
**Place of residence**																
Urban	1				1		1		1		1		1			
Rural	1.12 [0.82,1.52]	0.481			0.64 [0.48,0.86]	0.003	0.63 [0.47,0.85]	0.002	1.58 [1.26,1.98]	<0.001	1.39 [1.05,1.84]	0.02	1.50 [0.93,2.42]	0.094		
**Family size**																
2-4 members	1				1				1				1			
5-10 members	1.00 [0.76,1.31]	0.989			1.25 [0.96, 1.63]	0.1			1.07 [0.86, 1.32]	0.559			1.09 [0.71,1.67]	0.701		
>10 members	0.54 [0.28, 1.04]	0.065			1.34 [0.64,2.84]	0.439			1.36 [0.92,2.02]	0.125			0.94 [0.11, 7.92	0.953		
**Number of children < 5**																
1	1				1				1				1		1	
2 or more	1.01 [0.80,1.27]	0.966			1.13 [0.88, 1.46]	0.332			1.00 [0.84, 1.19]	0.971			1.51 [0.96, 2.37]	0.076	1.60 [1.02,2.50]	0.04
**Type of cooking fuels**																
Clean	1				1				1				1			
Unclean	0.22 [0.03, 1.45]	0.115			1.22 [0.48,3.12]	0.681			1.49 [1.19,1.87]	0.001			1			
**Household wealth quantile**																
Richest	1		1		1				1		1		1		1	
Fourth	1.06 [0.70, 1.60]	0.796	0.98 [0.65,1.49]	0.934	0.89 [0.60,1.32]	0.549			1.63 [1.22,2.18]	0.001	1.60 [1.12,2.2]	0.009	1 [0.52, 1.90]	0.99	0.82 [0.42, 1.59]	0.553
Middle	1.10 [.71, 1.71]	0.666	0.96 [0.61,1. 9]	0.848	1.20 [0.81,1.79]	0.358			2.13 [1.57,2.90]	<0.001	1.84 [1.24,2.72]	0.002	0.51 [0.24, 1.08]	0.078	0.34 [0.15, 0.77]	0.010
Poorer	1.3 [0.85, 2.00]	0.232	1.15 [0.77,1.72]	0.484	1.20 [0.86,1.66]	0.277			1.77 [1.27,2.45]	0.001	1.33 [0.87,2.04]	0.181	1.51 [0.80, 2.84]	0.206	1.38 [0.72, 2.64]	0.326
Poorest	2.36 [1.50, 3.72]	<0.001	2.19 [1.38,3.45]	0.001	1.14 [0.80, 1.63]	0.477			1.69 [1.18,2.41]	0.004	1.20 [0.76,1.91]	0.438	1.26 [0.62, 2.56]	0.515	1.13 [0.55, 2.31]	0.733
**Sources of drinking water**																
Improved	1				1				1				1			
Unimproved	0.96 [0.73, 1.25]	0.746			1.14 [0.57,2.30]	0.704			1.33 [1.09, 1.62]	0.006			0.80 [0.53, 1.21]	0.296		
**Type of toilet facility**																

Improved	1				1				1				1			
Unimproved	0.91 [0 .72,1.17]	0.474			1.37 [0.80, 2.35]	0.252			1.35 [1.09, 1.68]	0.007			0.97 [0.62, 1.52]	0.908		
**Illiteracy**																
Yes	1				1				1				1			
No	0.96 [0.70,1.31]	0.775			0.66 [0.35, 1.22]	0.182			1.32 [1.05, 1.67]	0.2			0.96 [0.55, 1.68]	0.883		
Care giver's related factors																
**Maternal level of education**																
Secondary and higher	1				1				1		1		1			
Primary	1.16 [0.81, 1.66]	0.418			1.36 [1.00, 1.84]	0.047			1.22 [0.97,1.53]	0.085	1.11 [0.87,1.42]	0.384	1.18 [0.66, 2.11]	0.578		
None	0.97 [0.64,1.46]	0.866			1.11 [0.79,1.55]	0.545			1.56 [1.23,1.97]	< 0.001	1.40 [1.07,1.83]	0.014	2.07 [0.98, 4.38]	0.058		
**Maternal age (years)**																
15-19 years	1				1				1							
20-34 years	0.96 [.59,1.58]	0.883			0.91 [0.51, 1.64]	0.762			0.77 [0.54,1.12]	0.172						
15-34 years													1			
35-49years	0.96 [.61,1.52]	0.867			0.94 [0.52,1.71]	0.842			0.85 [0.59,1.24]	0.401			1.29 [0.82, 2.02]	0.269		
**Paternal age (years)**																
18-34	1				1				1				1			
35-44	0.38 [0.04,3.58]	0.4			0.96 [0.74,1.24]	0.751			0.93 [0.75,1.16]	0.525			1.76 [1.06, 2.92]	0.028		
45+	0.32 [0.033,2.9]	0.314			0.79 [0.49, 1.28]	0.342			1.15 [0.94, 1.42]	0.176			1.40 [0.72, 2.72]	0.321		
**Maternal marital status**																
Married	1				1				1				1			
Unmarried	0.88 [0.62, 1.24]	0.458			1.73 [0.44, 6.79]	0.427			0.73 [0.42, 1.26]	0.259			1.34 [0.65, 2.78]	0.431		
**Maternal age at marriage**																
≤ 18 years	1				1				1				1			
>18 years	0.921 [.58, 1.45]	0.723			1.17 [0.86,1.58]	0.324			0.81 [0.68, 0.96]	0.016			1.34 [0.31, 5.85]	0.700		
**Maternal body mass index (BMI)**																
<18.5	1		1		1		1		1		1		1		1	
18.5-24.9	2.66 [2.07,3.41]	<0.001	2.91 [2.27, 3.73]	<0.001	2.01 [1.51, 2.68]	<0.001	2.10 [1.57, 2.80]	<0.001	1.80 [1.33, 2.43]	< 0.001	2.96 [2.07, 4.24]	<0.001	2.34 [1.50, 3.64]	<0.001	3.41 [2.11, 5.50]	<0.001

25+	8.91 [4.12,19.2]	<0.001	9.95 [4.43,22.4]	<0.001	2.29 [1.02, 5.13]	0.045	2.52 [1.06, 6,00]	0.037	0.67 [0.26, 1.71]	0.401	1.06 [0.34, 3.29]	0.918	10.21 [2.91, 35.69]	<0.001	15.94 [4.37, 58.14]	<0.001
**Listening to radio**																
No	1				1				1				1			
Yes	0.7 [0.56,1.06]	0.111			0.85 [0.62,1.15]	0.279			0.88 [0.73, 1.07]	0.205			0.95 [0.65, 1.40]	0.808		
**Watching TV**																
No	1				1				1				1			
Yes	0.60 [0.42, 0.87]	0.006			1.89 [0.61,5.88]	0.271			0.69 [0.55,0.87]	0.002			0.61 [0.21, 1.75]	0.355		
Child related factors																
**Child's sex**																
Boy	1		1		1				1		1		1			
Girl	0.50 [0.39, 0.64]	<0.001	0.50 [0.39,0.65]	<0.001	0.84 [0.65,1.10]	0.205			0.69 [0.58,0.82]	<0.001	0.63 [0.52, 0.76]	<0.001	1.09 [0.70, 1.69]	0.709		
**Child's age (months)**																
0-5	1		1		1		1		1		1		1		1	
6-11 m	0.67 [0.47, 0.96]	0.031	0.57 [0.39, 0.82]	0.003	1.04 [0.72,1.51]	0.84	0.96 [0.66,1.40]	0.844	1.71 [1.26,2.32]	0.001	1.84 [1.35, 2.51]	<0.001	0.59 [0.29, 1.19]	0.141	0.47 [0.23, 0.95]	0.03
12-17 m	0.86 [0.60,1.24]	0.422	0.77 [0.54, 1.10]	0.148	1.21 [0.84,1.73]	0.306	1.17 [0.82,1.68]	0.389	3.97 [2.92, 5.40]	<0.001	2.99 [1.96, 4.57]	<0.001	0.46 [0.24, 0.88]	0.02	0.36 [0.18, 0.72]	0
18-23	1.22 [0 .88,1.70]	0.231	1.22 [0.89,1.68]	0.219	1.52 [1.04, 2.23]	0.031	1.52 [1.026,2.3]	0.037	5.92 [4.38,7.10]	<0.001	4.33 [2.77, 6.80]	<0.001	0.91 [0.52,1.58]	0.726	0.85 [0.49,1.49]	0.58
**Child's birth order**																
1	1				1				1				1			
>2	0.92 [0.66,1.26]	0.59			0.91 [0.70,1.19]	0.476			1.02 [0.85, 1.21]	0.863			1.90 [0.93,3.7]	0.079		
Health facility access factors																
**Place of childbirth**																
Health facility	1				1				1				1			
Home	1.11 [0.53,2.32]	0.79			0.95 [0.67,1.34]	0.771			1.30 [1.02, 1.66]	0.033			1			
**Number of antenatal visits**																
8+	1				1				1				1			
4-7 visits	1.09 [0.72,1.67]	0.677			1.07 [0.82,1.38]	0.632			1.12 [0.76,1.63]	0.595			0.92 [0.53, 1.58]	0.752		
1-3 visits	1.27 [0.82,1.95]	0.28			1.01 [0.68,1.51]	0.954			1.24 [0.85,1.82]	0.264			0.74 [0.33, 1.65]	0.463		

None	0.97 [0.62,1.51]	0.882			0.99 [0.59, 1.66]	0.966			1.48 [0.98,2.23]	0.64			1.24 [0.62, 2.46]	0.542		
**Delivery assistants**																
Health professionals	1				1				1				1			
Non-Health professionals	0.74 [0.54,1.02]	0.065			0.89 [0.59,1.35]	0.588			1.18 [0.98,1.44]	0.083			1.04 [0.50, 2.16]	0.921		
**Type of delivery**																
Non-caesarean	1				1				1				1			
Caesarean	0.88 [0.66,1.17]	0.375			1.08 [0.83,1.40]	0.581			1.32 [0.97, 1.80	0.08			0.82 [0.53, 1.27]	0.378		
**Postnatal check up**																
0-2 days	1								1				1			
After 2	1.76 [1.02,3.04]	0.042			1.52 [0.77,2.99]	0.226			1.06 [0.41,2.72]	0.907			0.61 [0.27, 1.38]	0.237		
No	1.07 [0.71, 1.61]	0.759			1.20 [0.92, 1.57]	0.174			0.85 [0.66, 1.08]	0.187			0.76 [0.43, 1.36]	0.354		
Dietary intake																
**Dietary diversity**																
5+ food groups	1				1				1				1			
<5 food groups	1.19 [0.92,1.52]	0.178			0.83 [0.64,1.06]	0.14			0.91 [0.72, 1.15]	0.423			1.47 [0.93, 2.33]	0.101		
**Early Initiation of breastfeeding**																
After 1 hour	1				1				1				1			
Within 1 hour	1.08 [0 .86,1.34]	0.512			1.12 [0.89, 1.42]	0.341			0.94 [0.74,1.19]	0.597			1.18 [0.77, 1.82]	0.441		
**Duration of breastfeeding**																
Up to 12 months	1				1				1		1		1			
>12 months	1.10 [0.88,1.38]	0.403			1.30 [0.99, 1.71]	0.062			3.38 [2.81, 4.06]	<0.001	1.81 [1.26,2.60]	0.001	1.08 [0.71, 1.64]	0.731		
Child health																
**Perceived baby size**																
Average	1				1				1		1		1		1	
Small	1.45 [1.08,1.95]	0.013	1.50 [1.11, 2.01]	0.007	1.53 [1.19, 1.97]	0.001	1.57 [1.22,2.02]	0.001	1.43 [1.18,1.74]	<0.001	1.55 [1.26,1.91]	<0.001	1.94 [1.06, 3.55]	0.032	2.32 [1.25, 4.32]	0.008
Large	0.70 [0.47,1.04]	0.077	0.55 [0.37,0.82]	0.004	0.86 [0.48,1.54]	0.609	0.80 [0.46,1.42]	0.451	1.02 [0.81, 1.29]	0.851	0.89 [0.67, 1.17]	0.396	0.35 [0.16, 0.77]	0.009	0.35 [0.17, 0.75]	0.007

**Diarrhea**																
No	1	0.172			1	0.624			1	0.633			1	0.361		
Yes	0.64 [0.33, 1.22]				0.93 [0.71,1.23]				1.04 [0.87, 1.25]				1.37 [0.70, 2.69]			
**Cough**																
No	1	0.303			1	0.262			1	0.095			1	0.947		
Yes	1.17 [0 .87,1.58]				0.85 [0.64, 1.13]				1.18 [0.97, 1.44]				1.02 [0.61, 1.70]			
**Fever**																
No	1	0.583			1	0.423			1	0.691			1	0.945		
Yes	1.11 [0.76,1.61]				0.88 [0.63,1.21]				0.95 [0.77,1.19]				0.98 [0.56, 1.73]			
**Any infection**																
No	1	0.966			1	0.183			1	0.464			1	0.75		
Yes	1.01 [0.75,1.35]				0.85 [0.66,1.08]				1.07 [0.89, 1.28]				0.93 [0.61, 1.44]			

Table S4. Multivariable analysis of stunting among children (24-59 months) in four Northern African countries, Algeria 2018-19 (N= 16419), Egypt 2014 (N=5053), Sudan 2014 (N= 12841), and Tunisia 2017-18 (N= 3615)

	**Algeria 2018-19**				**Egypt 2014**				**Sudan 2014**				**Tunisia 2017-18**			
**Variable**	**OR (95%CI)**	**P-value**	**AOR (95%CI)**	**P-value**	**OR (95%CI)**	**P-value**	**AOR (95%CI)**	**P-value**	**OR (95%CI)**	**P-value**	**AOR (95%CI)**	**P-value**	**OR (95%CI)**	**P-value**	**AOR (95%CI)**	**P-value**
Household factors																
**Place of residence**																
Urban	1				1				1		1		1		1	
Rural	1.10 [0.87, 1.40]	0.405			0.99 [0.76,1.28]	0.921			1.87 [1.52, 2.31]	<0.001	1.71 [0.35,2.16]	<0.001	1.17 [0.73, 1.89]	0.505	1.29 [0.89, 1.89]	0.18
**Family size**																
2-4 members	1				1				1				1		1	
5-10 members	1.06 [0.86, 1.32]	0.574			0.98 [0.77, 1.24]	0.837			1.05 [0.89, 1.25]	0.557			1.10 [0.76, 1.58]	0.618	1.09 [0.82, 1.45]	0.556
>10 members	0.84 [0.50, 1.43]	0.527			1.18 [0.63, 2.19]	0.611			0.92 [0.71, 1.21]	0.563			0.73 [0.89, 6.04]	0.771	0.81 [0.18, 3.65]	0.78
**Number of children < 5**																
1	1				1				1				1			
2 or more	0.71 [0.58,0.87]	0.001			1.09 [0.86,1.38]	0.465			0.92 [0.80,1.05]	0.202			1.08 [0.79,1.48]	0.629		
**Type of cooking fuels**																
Clean	1				1				1				1			
Unclean	1.84 [0.82, 4.11]	0.136			0.64 [0.18,2.3]	0.491			1.33 [1.08, 1.65]	0.009			3.42 [0.41, 28.69]	0.257		
**Household wealth quantile**																
Richest	1				1		1		1		1		1			
Fourth	0.93 [0.66, 1.32]	0.704	0.93 [0.64, 1.34]	0.697	1.44 [0.96, 2.17]	0.078	1.70 [1.13, 2.55]	0.012	1.98 [1.58, 2.48]	<0.001	1.43 [1.12, 1.82]	0.004	0.93 [0.54, 1.59]	0.782		
Middle	0.70 [0.48, 1.01]	0.058	0.73 [0.50, 1.07]	0.109	1.72 [1.16, 2.54]	0.007	2.04 [1.40, 2.98]	<0.001	2.44 [1.90, 3.14]	<0.001	1.54 [1.17, 2.04]	0.002	1.09 [0.63, 1.88]	0.749		
Poorer	1.01 [0.71, 1.44]	0.94	1.02 [0.70, 1.47]	0.937	1.45 [0.97, 2.18]	0.072	1.68 [1.11, 2.54]	0.014	1.82 [1.39, 2.38]	<0.001	1.12 [0.81, 1.55]	0.481	1.05 [0.57, 1.93	0.881		
Poorest	1.38	0.076	1.46	0.043	1.79	<0.001	2.12	<0.001	1.54 [1.14, 2.09]	0.005	0.88	0.493	1.31 [0.67, 2.55]	0.432		

	[0.97, 1.96]		[1.01, 2.11]		[1.20, 2.67]		[1.43, 3.14]				[0.61, 1.27]					
**Sources of drinking water**																
Improved	1				1				1				1		1	
Unimproved	0.95 [0.73, 1.22]	0.667			0.74 [0.46, 1.19]	0.22			1.21 [1.01, 1.46]	0.041			1.61 [1.10, 2.36]	0.015	1.52 [0.83, 2.79]	0.176
**Type of toilet facility**																
Improved	1				1				1				1		1	
Unimproved	0.88 [0.70, 1.12]	0.302			0.58 [0.35, 0.94]	0.029			1.52 [1.26, 1.84]	<0.001			1.48 [1.00, 2.17]	0.048	0.95 [0.52, 1.74]	0.879
**Illiteracy**																
Yes	1				1				1				1			
No	0.92 [0.72, 1.17]	0.497			1.16 [0.70, 1.91]	0.57			1.10 [0.94, 1.28]	0.226			1.43 [0.88, 2.33]	0.146		
Care giver's related factors																
**Maternal level of education**																
Secondary and higher	1				1				1		1		1			
Primary	1.03 [0.78, 1.36]	0.823			0.87 [0.62, 1.23]	0.435			1.92 [1.55, 2.39]	<0.001	1.68 [0.32, 2.15]	<0.001	1.16 [0.80, 1.69]	0.431		
None	1.22 [0.88, 1.68]	0.229			1.10 [0.83, 1.45]	0.507			2.31 [1.83, 2.91]	<0.001	1.97 [1.48, 2.62]	<0.001	1.64 [0.85, 3.15]	0.141		
**Maternal age (years)**																
15-19 years	1				1				1							
20-34 years	0.90 [0.66, 1.22]	0.496			0.56 [0.15, 2.06]	0.383			1.00 [0.68, 1.49]	0.987						
15-34 years													1		1	
35-49years	0.85 [0.63, 1.14]	0.283			0.65 [0.17, 2.44]	0.524			0.89 [0.57, 1.41]	0.625			1.16 [0.84, 1.61]	0.369	1.34 [0.94, 1.92]	0.109
**Paternal age (years)**																
18-34	1				1		1		1				1			
35-44	0.04 [0.03, 0.45]	0.009			0.68 [0.54, 0.85]	0.001	0.73 [0.57, 0.94]	0.016	0.80 [0.68, 0.94]	0.007			1.35 [0.79, 2.31]	0.268		
45+	0.04	0.009			0.92	0.616	1.02	0.904	0.86 [0.72, 1.02]	0.089			0.95 [0.48, 1.87]	0.878		

	[0.03, 0.45]				[0.65, 1.29]		[0.72, 1.44]									
**Maternal marital status**																
Married	1				1				1				1			
Unmarried	1.18 [0.921,1.50]	0.195			0.88 [0.36, 2.18]	0.78			0.74 [0.54, 1.00]	0.05			1.18 [0.71, 1.94]	0.523		
**Maternal age at marriage**																
≤ 18 years	1				1				1		1		1			
>18 years	0.98 [0.69, 1.40]	0.912			1.23 [0.95, 1.59]	0.115			0.77 [0.67, 0.88]	<0.001	0.89 [0.77, 1.02]	0.091	1.10 [0.43, 2.81]	0.837		
**Maternal body mass index (BMI)**																
<18.5	1		1		1		1		1		1		1			
18.5-24.9	4.63 [3.64, 5.89]	<0.001	4.82 [3.73, 6.23]	<0.001	2.86 [2.22, 3.69]	<0.001	2.81 [2.16, 3.65]	<0.001	3.96 [2.41, 6.48]	<0.001	4.39 [2.49, 7.72]	<0.001	5.39 [3.75, 7.75]	<0.001	5.54 [3.82, 8.03]	<0.001
25+	8.14 [4.42,15.0]	<0.001	8.85 [4.76,16.47]	<0.001	3.34 [1.72, 6.49]	<0.001	2.90 [1.25, 6.69]	0.013	0.40 [0.18, 0.87]	0.02	0.43 [0.21, 0.90]	0.026	15.55 [6.82,35.5]	<0.001	12.96 [5.47,30.7]	<0.001
**Listening to radio**																
No	1				1				1				1			
Yes	0.83 [0.62, 1.10]	0.185			0.83 [0.61, 1.14]	0.256			0.76 [0.66, 0.87]	<0.001			0.75 [0.50, 1.12]	0.158		
**Watching TV**																
No	1				1				1				1			
Yes	0.75 [0.56, 1.00]	0.053			0.86 [0.42, 1.77]	0.678			0.60 [0.51, 0.70]	<0.001			0.57 [0.25, 1.30]	0.18		
Child related factors																
**Child's sex**																
Boy	1		1		1				1				1			
Girl	0.76 [0.62, 0.94]	0.011	0.75 [0.61, 0.93]	0.008	0.98 [0.77, 1.25]	0.869			0.93 [0.83, 1.03]	0.173			1.21 [0.85, 1.71]	0.282		
**Child's age (months)**																
24-29	1		1		1		1		1		1		1		1	
30-35	1.19 [0.85, 1.67]	0.302	1.42 [0.97, 2.08]	0.07	1.30 [0.94, 1.80]	0.108	1.44 [1.01, 2.05]	0.044	1.14 [0.93, 1.40]	0.206	1.17 [0.94, 1.45]	0.155	0.90 [0.52, 1.58]	0.724	1.27 [0.72, 2.25]	0.415
36-41	0.83	0.329	0.99	0.979	0.44	<0.001	0.51	0	0.71	0,001	0.73	0.002	0.55	0.103	0.74	0.405

	[0.57, 1.21]		[0.66, 1.50]		[0.31, 0.62]		[0.36, 0.74]		[0.58, 0.87]		[0.59, 0.89]		[0.27, 1.13]		[0.36, 1.51]	
42-47	1.02 [0.70, 1.48]	0.924	1.38 [0.93, 2.06]	0.11	0.53 [0.38, 0.72]	<0.001	0.62 [0.45, 0.85]	0.003	1.12 [0.92, 1.36]	0.252	1.13 [0.93, 1.37]	0.22	0.98 [0.56, 1.70]	0.942	1.37 [0.78, 0.43]	0.276
48-53	0.71 [0.47, 1.07]	0.103	0.96 [0.63, 1.46]	0.836	0.41 [0.29, 0.58]	<0.001	0.48 [0.33, 0.70]	<0.001	0.63 [0.51, 0.77]	<0.001	0.66 [0.53, 0.83]	<0.001	0.50 [0.26, 0.93]	0.03	0.76 [0.40, 1.43]	0.39
54-59	1.09 [0.74, 1.62]	0.662	1.56 [1.02, 2.37]	0.041	0.26 [0.17, 0.42]	<0.001	0.31 [0.19, 0.51]	<0.001	0.67 [0.54, 0.82]	<0.001	0.71 [0.57, 0.88]	0.002	0.64 [0.37, 1.11]	0.115	0.84 [0.48, 1.46]	0.537
**Child's birth order**																
1	1				1				1				1			
>2	1.36 [1.06, 1.75]	0.016			1.10 [0.86, 1.40]	0.468			0.94 [0.83, 1.07]	0.377			1.02 [0.61, 1.72]	0.939		
Health facility access factors																
**Postnatal check up**																
0-2 days	1				1				1				1			
After 2	0.89 [0.34, 2.35]	0.818			0.67 [0.26, 1.69]	0.392			0.63 [0.16, 2.48]	0.508			1.30 [0.28, 6.02]	0.739		
No	1.14 [0.71, 1.84]	0.583			0.90 [0.68, 1.18]	0.44			1.24 [0.90, 1.72]	0.185			2.08 [0.67, 6.43]	0.203		
**Diarrhea**																
No	1				1				1				1			
Yes	0.70 [0.42, 1.18]	0.182			1.17 [0.87, 1.58]	0.293			1.40 [1.18, 1.65]	<0.001	1.39 [1.18, 1.64]	<0.001	0.80 [0.38, 1.65]	0.542		
**Cough**																
No	1				1				1				1		1	
Yes	1.09 [0.83, 1.42]	0.538			0.92 [0.72, 1.17]	0.486			1.03 [0.90, 1.18]	0.687			0.70 [0.44, 1.11]	0.13	0.95 [0.44, 2.04]	0.893
**Fever**																
No	1				1				1				1			
Yes	1.02 [0.77, 1.34]	0.905			0.90 [0.68, 1.20]	0.462			1.17 [0.98,1.39]	0.074			0.62 [0.42,0.91]	0.015		
**Any infection**																
No	1				1				1				1		1	
Yes	1.05 [0.83, 1.32]	0.692			0.97 [0.77, 1.22]	0.784			1.13 [0.98, 1.31]	0.088			0.60 [0.42, 0.85]	0.005	0.69 [0.35, 1.38]	0.298

Table S5. Multivariable analysis of stunting among children (0-59 months) in Northern Africa, Algeria 2018-19 (N= 16419), Egypt 2014 (N=5053), Sudan 2014 (N= 12841), and Tunisia 2017-18 (N= 3615)

	**Algeria 2018-19**				**Egypt 2014**				**Sudan 2014**				**Tunisia 2017-18**			
**Variable**	**OR (95%CI)**	**P-value**	**AOR (95%CI)**	**P-value**	**OR (95%CI)**	**P-value**	**AOR (95%CI)**	**P-value**	**OR (95%CI)**	**P-value**	**AOR (95%CI)**	**P-value**	**OR (95%CI)**	**P-value**	**AOR (95%CI)**	**P-value**
Household factors																
**Place of residence**																
Urban	1	0.313			1	0.064			1	<0.001	1	0.001	1	0.18		
Rural	1.11 [0.91, 1.35]				0.82 [0.67, 1.01]				1.74 [1.46, 2.07]		1.43 [1.16, 1.77]		1.29 [0.89, 1.89]			
**Family size**																
2-4 members	1				1				1				1			
5-10 members	1.04 [0.88, 1.24]	0.625			1.08 [0.90, 1.30]	0.412			1.14 [1.01, 1.28]	0.038			1.09 [0.82, 1.45]	0.556		
>10 members	0.70 [0.46, 1.06]	0.091			1.23 [0.72, 2.11]	0.453			1.12 [0.92, 1.36]	0.257			0.81 [0.18, 3.65]	0.78		
**Number of children < 5**																
1	1	0.009			1	0.273			1	0.256			1	0.137		
2 or more	0.80 [0.68, 0.95]				1.10 [0.92, 1.32]				0.94 [0.85, 1.04]				1.23 [0.94, 1.61]			
**Type of cooking fuels**																
Clean	1	0.411			1	0.828			1	<0.001			1	0.36		
Unclean	1.40 [0.63, 3.12]				0.92 [0.44, 1.92]				1.39 [1.18, 1.64]				2.22 [0.40, 12.25]			
**Household wealth quantile**																
Richest	1		1		1		1		1		1		1			
Fourth	0.97 [0.74,1.27]	0.825	0.97 [0.74,1.26]	0.799	1.14 [0.84,1.54]	0.396	1.22 [0.90,1.66]	0.199	1.81 [1.51,2.17]	<0.001	1.56 [1.07,2.27]	0.021	0.96 [0.63,1.46]	0.837		
Middle	0.82 [0.61,1.10]	0.179	0.75 [0.56,1.01]	0.057	1.44 [1.08,1.93]	0.013	1.60 [1.20,2.13]	0.001	2.26 [1.84,2.77]	<0.001	1.69 [1.15,2.49]	0.008	0.87 [0.56,1.34]	0.516		
Poorer	1.09 [0.83,1.45]	0.532	0.95 [0.71,1.26]	0.704	1.30 [1.00,1.70]	0.054	1.48 [1.14,1.93]	0.003	1.78 [1.42,2.23]	<0.001	1.28 [0.85,1.93]	0.234	1.21 [0.75,0.95]	0.430		
Poorest	1.66 [1.25,2.20]	<0.001	1.44 [1.06,1.96]	0.02	1.44 [1.09,1.90]	0.011	1.67 [1.27,2.21]	<0.001	1.61 [1.23,2.11	0.001	1.08 [0.69,1.68]	0.732	1.29 [0.77,2.16]	0.326		
**Sources of drinking water**																
Improved	1	0.612			1	0.643			1	0.002			1	0.158		
Unimproved	0.95 [0.78, 1.159]				0.89 [0.56, 1.43]				1.27 [1.09, 1.48]				1.23 [0.92, 1.64]			
**Type of toilet facility**																

Improved	1				1				1				1			
Unimproved	0.89 [0.75, 1.07]	0.23			0.92 [0.66, 1.28]	0.612			1.46 [1.23,1.73]	<0.001			1.25 [0.93, 1.68]	0.142		
**Illiteracy**																
Yes	1				1				1				1			
No	0.92 [0.76, 1.12]	0.41			0.91 [0.61, 1.37]	0.66			1.19 [1.04, 1.36]	0.013			1.20 [0.85, 1.70]	0.297		
Care giver's related factors																
**Maternal level of education**																
Secondary and higher	1				1				1		1		1			
Primary	1.08 [0.86, 1.37]	0.49			1.09 [0.88, 1.36]	0.438			1.63 [1.38, 1.94]	<0.001	1.27 [1.02, 1.59]	0.032	1.16 [0.83, 1.62]	0.375		
None	1.14 [0.88, 1.49]	0.322			1.10 [0.88, 1.37]	0.392			2.05 [1.69, 2.48]	<0.001	1.49 [1.19, 1.86]	0.001	1.78 [1.09, 2.89]	0.021		
**Maternal age (years)**																
15-19 years	1				1				1							
20-34 years	0.89 [0.68,1.16]	0.398			0.81 [0.49, 1.36]	0.429			1.04 [0.82, 1.33]	0.727						
15-34 years													1			
35-49years	0.88 [0.68, 1.12]	0.295			0.89 [0.53, 1.50]	0.663			1.05 [0.80, 1.38]	0.709			1.19 [0.91, 1.56]	0.197		
**Paternal age (years)**																
18-34	1		1		1		1		1				1		1	
35-44	0.15 [0.05, 0.49]	0.002	0.22 [0.07,0.70]	0.01	0.78 [0.66, 0.93]	0.006	0.79 [0.66, 0.94]	0.01	0.89 [0.78, 1.02]	0.103			1.51 [1.03, 2.22]	0.034	1.53 [1.03, 1.26]	0.036
45+	0.14 [0.04, 0.48]	0.002	0.19 [0.06,0.63]	0.007	0.88 [0.67, 1.15]	0.337	0.89 [0.67, 1.19]	0.434	1.05 [0.91, 1.20]	0.535			1.10 [0.66, 1.82]	0.709	1.21 [0.73, 2.01]	0.449
**Maternal marital status**																
Married	1				1				1				1			
Unmarried	1.09 [0.88,1.34]	0.433			1.03 [0.47,2.25]	0.934			0.80 [0.61, 1.04]	0.099			1.22 [0.81,1.86]	0.344		
**Maternal age at marriage**																
≤ 18 years	1				1		1		1		1		1			
>18 years	0.96 [*0.76*, 1.26]	0.756			1.20 [1.00, 1.44]	0.046	1.24 [1.04, 1.49]	0.019	0.78 [0.70, 0.87]	<0.001	0.85 [0.75, 0.96]	0.01	1.18 [0.54, 2.54]	0.68		
**Maternal body mass index (BMI)**																
<18.5	1		1		1		1		1		1		1		1	

18.5-24.9	3.34 [2.78, 4.00]	<0.001	3.81 [3.07,4.73]	<0.001	2.39 [1.96, 2.91]	<0.001	2.47 [2.02, 3.02]		2.14 [1.62, 2.84]	<0.001	2.11 [1.58, 2.82]	<0.001	3.74 [2.86, 4.89]	<0.001	3.77 [2.83, 5.01]	<0.001
25+	8.34 [5.07, 13.73]	<0.001	8.77 [5.15,14.94]	<0.001	2.75 [1.63, 4.63]	<0.001	2.79 [1.64, 4.75]	<0.001	0.48 [0.27, 0.87]	0.015	0.34 [0.17, 0.68]	0.002	13.21 [6.81,25.64]	<0.001	13.63 [6.76, 27.49]	<0.001
**Listening to the radio**																
No	1	0.042			1	0.108			1	<0.001			1	0.147		
Yes	0.80 [0.65,0.99]				0.84 [0.68,1.04]				0.80 [0.72,0.89]				0.82 [0.62,1.07]			
**Watching TV**																
No	1	0.002			1	0.64			1	<0.001			1	0.155		
Yes	0.69 [0.55, 0.87]				1.16 [0.62, 2.19]				0.63 [0.55, 0.73]				0.58 [0.28,1.23]			
Child related factors																
**Child's sex**																
Boy	1	<0.001	1	<0.001	1	0.317			1		1	0.001	1	0.292		
Girl	0.66 [0.56, 0.77]		0.67 [0.56, 0.80]		0.92 [0.77, 1.09]				0.84 [0.77, 0.91]	<0.001	0.84 [0.77, 0.93]		1.16 [0.88, 1.52]			
**Child's age (months)**																
**0-23 m**	1	0.056	1	0.003	1	0.189	1	0.635	1		1	<0.001	1	0.596	1	0.271
**24-59m**	1.19 [1.00, 1.43]		1.42 [1.13, 1.79]		0.89 [0.75,1.06]		1.04 [0.87, 1.25]		1.88 [1.67, 2.11]	<0.001	2.01 [1.77, 2.27]		0.93 [0.70, 1.23]		1.18 [0.88, 1.60]	
**Child's birth order**																
1	1	0.068			1	0.918			1	0.995			1	0.261		
>2	1.20 [0.99, 1.47]				0.99 [0.81, 1.21]				1.00 [0.90, 1.11]				1.28 [0.83, 1.96]			
Health facility access factors																
**Postnatal check up**																
0-2 days	1				1				1				1			
After 2	1.46 [0.89, 2.39]	0.13			1.05 [0.68, 1.60]	0.833			0.84 [0.36, 1.96]	0.682			0.74 [0.36, 1.50]	0.397		
No	1.17 [0.85, 1.60]	0.329			1.02 [0.84, 1.25]	0.829			1.13 [0.91, 1.41]	0.254			0.96 [0.59, 1.56]	0.872		
Child health																
**Diarrhea**																
No	1	0.038			1	0.607			1	0.019	1	<0.001	1	0.656		
Yes	0.64 [0.42, 0.98]				1.05 [0.87, 1.27]				1.15 [1.02, 1.30]		1.37 [1.19, 1.57]		1.12 [0.68,1.85]			
**Cough**																
No	1	0.31			1	0.151			1	0.086			1	0.097		
Yes	1.11				0.89				1.11				0.76			

	[0.90, 1.37]				[0.75, 1.045]				[0.99, 1.24]				[0.55, 1.05]			
**Fever**																
No	1				1				1	0.437			1			
Yes	1.05 [0.83, 1.32]	0.69			0.89 [0.72, 1.10]	0.294			1.06 [0.91, 1.24]				0.81 [0.56, 1.17]	0.27		
**Any infection**																
No	1				1				1				1			
Yes	1.03 [0.84, 1.25]	0.797			0.92 [0.78, 1.08]	0.317			1.09 [0.96, 1.23]	0.187			0.73 [0.55, 0.96]	0.023		
